# Targeting *SRSF2* mutations in leukemia with RKI-1447: A strategy to impair cellular division and nuclear structure

**DOI:** 10.1016/j.isci.2024.109443

**Published:** 2024-03-06

**Authors:** Minhua Su, Tom Fleischer, Inna Grosheva, Melanie Bokstad Horev, Malgorzata Olszewska, Camilla Ciolli Mattioli, Haim Barr, Alexander Plotnikov, Silvia Carvalho, Yoni Moskovich, Mark D. Minden, Noa Chapal-Ilani, Alexander Wainstein, Eirini P. Papapetrou, Nili Dezorella, Tao Cheng, Nathali Kaushansky, Benjamin Geiger, Liran I. Shlush

**Affiliations:** 1 State Key Laboratory of Experimental Hematology, National Clinical Research Center for Blood Diseases, Haihe Laboratory of Cell Ecosystem, Institute of Hematology & Blood Diseases Hospital, Chinese Academy of Medical Sciences & Peking Union Medical College, Tianjin, China; 2 Department of Molecular and Cellular Biology, Weizmann Institute of Science, Rehovot, Israel; 3 Department of Immunology and Regenerative Biology, Weizmann Institute of Science, Rehovot, Israel; 4 Department of Oncological Sciences, Icahn School of Medicine at Mount Sinai, New York, NY, USA; 5 Wohl Institute for Drug Discovery, Grand Israel National Center for Personalized Medicine, Weizmann Institute of Science, Rehovot, Israel; 6 Princess Margaret Cancer Centre, University Health Network (UHN), Toronto, ON Canada; 7 Molecular Hematology Clinic, Maccabi Healthcare, Tel Aviv, Israel; 8 Division of Hematology, Rambam Healthcare Campus, Haifa, Israel; 9 Electron Microscopy Unit, Weizmann Institute of Science, Rehovot, Israel

**Keywords:** Molecular biology, Cell biology, Cancer

## Abstract

Spliceosome machinery mutations are common early mutations in myeloid malignancies; however, effective targeted therapies against them are still lacking. In the current study, we used an *in vitro* high-throughput drug screen among four different isogenic cell lines and identified RKI-1447, a Rho-associated protein kinase inhibitor, as selective cytotoxic effector of *SRSF2* mutant cells. RKI-1447 targeted *SRSF2* mutated primary human samples in xenografts models. RKI-1447 induced mitotic catastrophe and induced major reorganization of the microtubule system and severe nuclear deformation. Transmission electron microscopy and 3D light microscopy revealed that *SRSF2* mutations induce deep nuclear indentation and segmentation that are apparently driven by microtubule-rich cytoplasmic intrusions, which are exacerbated by RKI-1447. The severe nuclear deformation in RKI-1447-treated *SRSF2* mutant cells prevents cells from completing mitosis. These findings shed new light on the interplay between microtubules and the nucleus and offers new ways for targeting pre-leukemic *SRSF2* mutant cells.

## Highlights

• RKI-1447 identified in high-throughput screening as potent for *SRSF2* mutant cells
• RKI-1447 effective in xenograft model against primary human leukemia samples
• The Inhibitor causes mitotic catastrophe, cytoskeleton reorganization, severe nuclear deformation
• The results point to a new therapeutic approach for leukemias involving *SRSF2* mutations

## Graphical abstract

**Figure:**
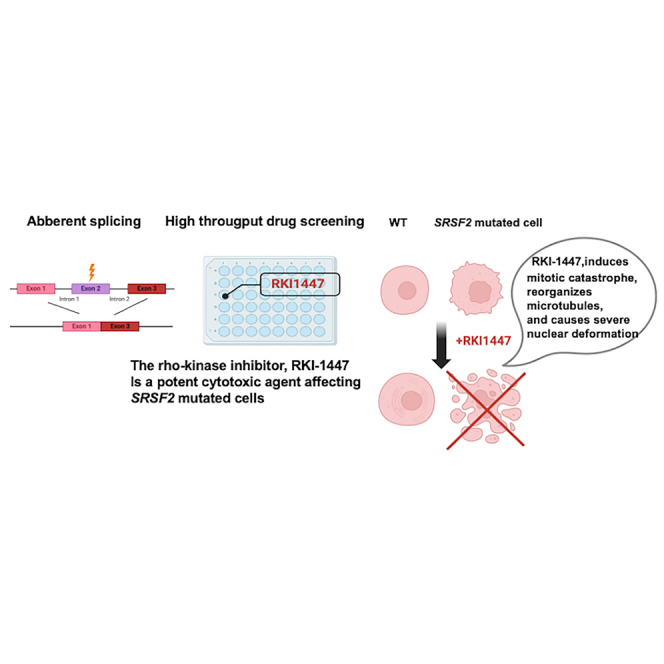

## Introduction

*SRSF2* mutations are the most common splicing machinery mutations (SMMs) identified in various myeloid malignancies (acute myeloid leukemia [AML], myelodysplastic syndrome [MDS], chronic myelomonocytic leukemia [CMML], and myeloproliferative neoplasms [MPNs]). Furthermore, it has been discovered that SMMs can be identified among healthy individuals as part of age-related clonal hematopoiesis (CH).^1^ The presence of age-related *SRSF2* mutations defines individuals with a high risk to progress to overt AML.^1^^,^^2^ The fact that *SRSF2* mutations are the first events in a high-risk leukemogenic process highlights their potential as targets for preventing leukemia. Accordingly, the next crucial step toward preventive therapy is the creation of an arsenal of safe interventions for targeting human hematopoietic cells carrying *SRSF2* mutations.

*SRSF2* is an RNA-binding protein that plays important roles in splicing of mRNA precursors. It was reported that *SRSF2* mutations change the RNA binding specificity of *SRSF2*, resulting in skipped/included exons. Specific motifs, GGNG and CCNG, were found to be enriched in the excluded exons and included exons, respectively.^3^^,^^4^ SMMs are commonly heterozygous, likely due to the need for a functioning wild-type (WT) allele, and are less tolerant to pharmacological inhibition of the splicing machinery.^5^ This rationale was successfully tested for one such splicing inhibitor, E7107, which decreased leukemia burden in mice carrying *SRSF2* mutations.^6^ However, a phase-I clinical study in which E7107 was administered to patients with myeloid malignancies was prematurely discontinued due to severe adverse effects.^7^ H3B-8800, an orally available analog of E7107, has been safely tested on MDS, AML, and CMML patients but did not induce partial/complete response in such patients.^8^ Others attempted to target SMMs by identifying specific pathogenic aberrant isoforms.^9^^,^^10^ Notably, *SRSF2* mutations are causing more than just mis-splicing errors; previous studies demonstrated that *SRSF2* mutations induce R-loops, DNA replication stress, and G2/M cell-cycle arrest.^11^ A functional CRISPR screen performed on Hoxb8 immortalized cell lines from R26-CreERT2 Srsf2P95H/+ mice indicated that cell-cycle pathways are probably important for cells carrying *SRSF2* mutations;^12^ however, so far, none of these approaches demonstrated clear clinical benefit.

In the current study, we used an unbiased approach, performing a high-throughput drug screening (HTDS) on four different *SRSF2*-mutated cell lines. Our results indicate that RKI-1447, a Rho-associated protein kinase inhibitor (ROCKi), can inhibit the growth of *SRSF2*-mutated AML cells *in vitro* and decrease the engraftment of primary *SRSF2*-mutated AML cells *in vivo*. ROCKs are effectors of the RhoA GTPase and have pleiotropic effects on cell growth, cell morphology and cytoskeletal organization and mechanics, through the phosphorylation of downstream targets.^13^^,^^14^ Notably, the cytoskeleton is a key player in regulating cell division, morphogenesis, and migration and has major effects on nuclear morphology.^15^^,^^16^^,^^17^^,^^18^ In this study, we show that RKI-1447 accentuated the deformed cytoskeletal-nuclear phenotype in *SRSF2*-mutated cells and induces deep nuclear indentations and segmentations, eventually leading to mitotic catastrophe and cell death.

## Results

### The establishment of *SRSF2*-mutated cell lines

**Figure 1:**
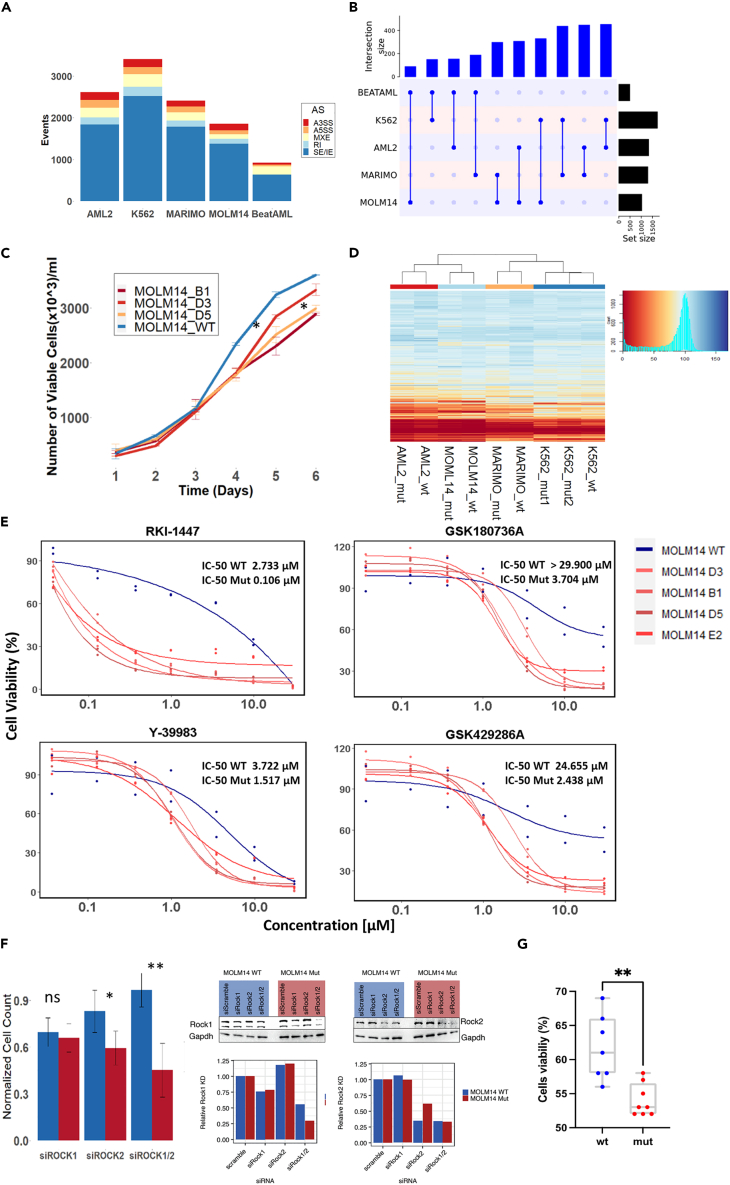
Establishment of *SRSF2* Mut cell line models and drug screen (A) Number of aberrantly spliced events in all *SRSF2* Mut lines (AML2, K562, MARIMO, MOLM14), and in Beat AML *SRSF2* Mut samples (N = 36) in comparison to *SRSF2* WT samples. Alternative 3′ splice sites (A3SS), alternative 5′ splice sites (A5SS), mutually exclusive exons (MXE), retained intron (RI), and skipped/included exons (SE/IE). (B) Overlap of aberrant exon spliced genes of *SRSF2* Mut cell lines and Beat AML database. Number of overlapped aberrant exon spliced genes identified in MOLM14, MARIMO, OCI-AML2, K562 *SRSF2* Mut cells and *SRSF2* Mut samples in Beat AML database were plotted by UpSetR (ver.1.4.0) and ComplexHeatmap (ver. 2.2.0). The numbers of aberrant exon spliced genes overlap between different cells lines and primary AML samples were shown. See also Figure S1A. (C) MOLM14 *SRSF2* Mut cells (B1, D3, D5) showed consistently lower growth rates than WT. The slower growth rate was significant after the fifth day in culture. Statistical analysis was performed using two-sided t tests; ∗p < 0.05. (D) Heatmap of cell viability for different isogenic *SRSF2* Mut cell lines and their WT controls after 48 h of exposure to 3,988 compounds (10 μM). Blue represents high cell viability, and red represents low cell viability. (E) Dose respond curve of WT and *SRSF2* Mut MOLM14 cell lines (four different biological replicates) with four different ROCKi compounds (RKI-1447, GSK180736A, Y-39983, and GSK429286A) at concentration of 0.037, 0.129, 0.369, 0.997, 3.49, 9.97, and 29.900 μM. (F) Cell counts 48 h after transfection with siRNA targeting ROCK1 (250 nM) or ROCK2 (250 nM) on MOLM14 WT and *SRSF2* Mut cells. Cell counts are normalized to non-targeting siRNA controls from each group. A significant reduction in cell viability was noted after knocking down ROCK2 or knocking down both ROCK1 and ROCK2 (250nM). 48 h after siRNA treatment, cells were harvested and processed for western blot as described in supplementary methods. Gapdh was used as internal control. (G) Isogenic *SRSF2* WT and P95L iPSC-derived HSPCs were cultured with DMSO or RKI-1447 (3 μM) for 48 h, and viability was measured with CellTiter-Glo, normalized to DMSO-treated in each group. The statistic was done with Mann-Whitney test, ∗p < 0.05; ∗∗p < 0.005; ∗∗∗p < 0.0005.

In the current study, we adopted an unbiased approach and conducted an HTDS using three commercially available compound libraries. Toward this goal, we introduced mutations in the *SRSF2* hotspot region around P95 by the CRISPR-Cas9 system into four hematopoietic cell lines (MOLM14, K562, MARIMO, and OCI-AML2) (Data S1). Splicing analysis of the engineered *SRSF2* mutated (Mut) lines replicated the aberrant splicing phenotype observed in primary AML samples, which is dominated by abnormal exon usage (Figure 1A, Data S2). Each one of the cell lines shared ∼150 genes which had abnormal exon usage with primary AML samples from the Beat AML^19^ (∼30% overlap) (Figure 1B). The overlap between genes with splice variants found in the Beat AML dataset and the four isogenic *SRSF2* Mut cell lines was statistically significant (p < 10^−6^ Figure S1A, Data S3). These results suggest that the isogenic *SRSF2* Mut cell lines successfully recapitulated primary *SRSF2* Mut AML aberrant splicing and can serve as a model for synthetic lethality screen.

Another known phenotypic consequence of *SRSF2* mutations is a decrease in growth rate of mutant versus WT cells.^11^ To validate these results, we studied the growth kinetics of our isogenic cell lines and observed a slower growth rate in OCI-AML2 and MOLM14 mutated lines compared to their isogenic WT controls (Figures 1C and S1B).

### *In vitro* HTDS reveals differential inhibition of *SRSF2* Mut cells by ROCKi

Having validated the functional consequences of *SRSF2* mutations in our cell lines, we next performed an HTDS of 3,988 chemical compounds administrated in a single dose (10 μM). In the majority of cases, both *SRSF2* Mut and WT cell lines responded similarly (Figure 1D, Data S4). In addition, OCI-AML2 and MOLM14 shared a similar drug response (Figure 1D). Following our primary screening, we identified compounds that were more cytotoxic to mutant versus WT cells (defined as a difference in viability greater than 10%, in at least two lines). A seven-dose half-maximal inhibitory concentration (IC50) assay was performed on three isogenic cell lines, and for each one of them (MOLM14, OCI-AML2, and MARIMO) at least three biological replicates of mutant clones were tested with the selected compounds (N = 44 compounds, Data S5). Four of these 44 compounds were ROCK inhibitors (ROCKis). The IC50 analysis revealed that MOLM14 *SRSF2* Mut lines were significantly more sensitive to four different ROCKis, including GSK429286A, GSK180736A, Y-39983, and specifically RKI-1447 (Figure 1E, Data S6). Some of the OCI-AML2 *SRSF2* Mut lines were also more sensitive to some of the ROCKis (Figure S1C). No response to ROCKi was noted in the MARIMO lines (Figure S1D). Based on this result, we chose to focus on RKI-1447 for following experiments on MOLM14 and OCI-AML2. To validate that RKI-1447 inhibits the ROCK's pathway in our system, we performed phosphoproteomics on MOLM14 and OCI-AML2 cells before and after 2- and 8-h exposure to RKI-1447. We demonstrated inhibited phosphorylation of *MYPT1*, a downstream target of ROCKs pathway (Figures S2A and S2B, Data S7), as has been demonstrated by others.^20^ We also demonstrated reduced phosphorylation of other downstream targets of ROCKs (Figures S2A and S2B). In order to clarify the role of ROCK1 and ROCK2 in the system, we knocked down ROCK1 and/or ROCK2 using a commercial small interfering RNA (siRNA) pool. Cell count was assessed 48 h after nucleofection. A significantly lower cell count was observed compared to WT controls after silencing ROCK2 or both ROCK1 and ROCK2 in MOLM14 Mut cells (Figure 1F). To further validate the efficacy of RKI-1447 against human pre-Leukemic hematopoietic stem cells (preL-HSPCs) *in vitro*, we exposed preL-HSPCs derived through *in vitro* differentiation from an iPSC line carrying *SRSF2* P95L and isogenic control WT induced pluripotent stem cells (iPSC)-HSPCs to RKI-1447.^9^^,^^21^ *SRSF2* Mut cells were more sensitive to RKI-1447 (Figures 1G and S2C). Taken together, we established functionally validated *SRSF2* Mut models. The HTDS identified four different drugs known to be ROCKi which introduced synthetic lethality to *SRSF2* Mut cell lines and was effective against iPSC line carrying *SRSF2* Mut. To further validate the relevance of these results in a more clinically relevant setting, we next tested the effect of RKI-1447 on primary human cells carrying *SRSF2* mutations *in vivo*.

### *In vivo* differential efficacy and safety of RKI-1447

**Figure 2:**
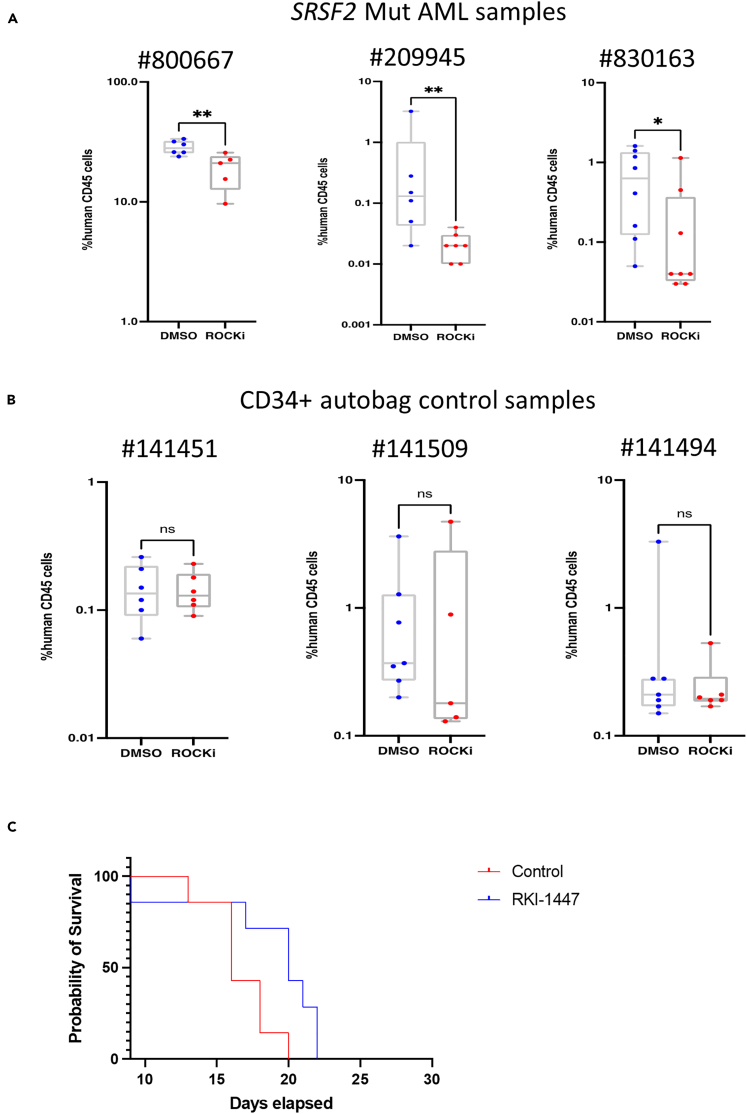
*in vivo* efficacy and toxicity of RKI-1447 (A) CD3-depleted frozen PBMC from three *SRSF2*-mutated acute myeloid leukemia (AML) samples were injected into SGM3 (#800667) or NSG mice (#209945 and #830163) intrafemorally (i.f.); after 5 weeks transplantation mice were treated with RKI-1447 (50 mg/kg) or DMSO control for 21 days. (B) NSG mice (n = 5–10/sample) were injected with 80,000 to 150,000 CD34^+^ cells from three mobilized peripheral blood samples (i.f.). On day 35 the animals were randomized to RKI-1447 or a carrier control. RKI-1447 was administered i.p. at a dose of 50 mg/kg daily for 21 days. On day 56 mice were sacrificed and analyzed for human CD45^+^ (hCD45) cells engraftment by flow cytometry. Mann-Whitney U test with FDR correction for multiple hypothesis testing, ∗p < 0.05; ∗∗p < 0.005; ∗∗∗p < 0.0005. (C) 5 ∗ 10ˆ6 MOLM14 mutated cells were injected into 225 rad irradiated NSG mice. The mice were treated with RKI-1447 (50 mg/kg/day), starting from day 3 following cell transplantation. The mice were treated every day via intraperitoneal (i.p.) injection for 21 days. The Kaplan-Meier test p = 0.01.

To study the efficacy of RKI-1447 on human samples, we first identified a dose and schedule that were efficacious in *in vivo* model of MOLM14 *SRSF2* Mut cells in NOD scid gamma (NSG) mice; engrafted animals were treated with different dosage of RKI-1447 (Figures S2D and S2E). Having established a non-toxic and efficacious dosage, we conducted similar experiments using engrafting primary AML patient samples that harbored *SRSF2* mutations (Figure 2A, Data S8). We isolated human CD45^+^ cells from the mice bone marrow and performed targeted sequencing of the *SRSF2* P95 region to confirm the engraftment of *SRSF2* Mut cells (Figure S3A). RKI-1447 significantly reduced engraftment compared to the control (DMSO-treated) group and inhibited both myeloid and multi-lineage engraftment of *SRSF2*-mutated AML samples (Figure S3B, Data S8), while RKI-1447 had no significant effect on *SRSF2* WT primary AML samples (Figure S3C, Data S8). In addition to the *SRSF2* WT AML samples, we also performed *in vivo* experiments on *SRSF2* WT CD34^+^ cells from three mobilized peripheral blood samples (Figure 2B); RKI-1447 had no significant inhibiting effect on the engraftment of these cells. Furthermore, the treatment with RKI-1447 resulted in survival benefits for mice engrafted with MOLM14 *SRSF2*-mutated cells (Figure 2C).

Once we established that RKI-1447 was active against human samples carrying *SRSF2* Mut, we aimed at testing its toxicity against different human tissues *in vitro*. We isolated human CD34^+^ HSPCs, seeded them in 384-well plates, and induced differentiation toward myeloid, erythroid, and megakaryoid lineages. RKI-1447 was added after seven days, and the cells were further incubated for 48 h, at which time cell viability was measured. RKI-1447 demonstrated limited toxicity to the three normal lineages (Figure S3D). Flow cytometry analysis of colonies after RKI-1447 treatment demonstrated that there were less percentage of CD34^+^ and CD11b+ cells under myeloid lineage differentiation condition (Figures S3E and S3F), but differentiation was normal toward erythrocytes and megakaryocytes (Figure S3G), and other myeloid populations.

Liver metabolism assay revealed that RKI-1447 is metabolized in the liver with a half-life of 11.42 min (Figure S3H) *in vitro*; hepatotoxicity was comparable to tamoxifen effect *in vitro* (Data S9). We also evaluated potential cardiotoxicity with the human ether a - go-go related gene (hERG) assay (a predictor assay based on a compound binding to hERG channel, which can lead to cardiotoxicity). Compared with Cisapride, a known cardiotoxic compound, RKI-1447 was predicted to have very minor cardiotoxicity (Figure S4A). To test whether the compound induced differentiation of our MOLM14 *SRSF2* Mut model, we performed flow cytometry for differentiation markers before and after 24 h of exposure to RKI-1447 (0.5 μM), and no difference in differentiation markers between treated and non-treated cells was noted (Figures S4B‒S4E). RNA sequencing (RNA-seq) analysis of the cells revealed that neutrophil differentiation-related genes *AZU1*, *CTSG*, *ELANE*, and *MPO* were not increased in level after exposure to RKI-1447 in *SRSF2* Mut MOLM14 cells (Figure S4F); in fact, the expression of *CTSG* and *ELANE* was downregulated in *SRSF2* WT after exposure to RKI-1447 (Figure S4G). Overall, we have shown efficacy and safety of RKI-1447 in *SRSF2*-mutated AML samples using patient derived xenograft (PDX) models and toxicity assays. Next, we aimed at understanding its mechanisms of action.

### RKI-1447-driven mitotic stress

**Figure 3:**
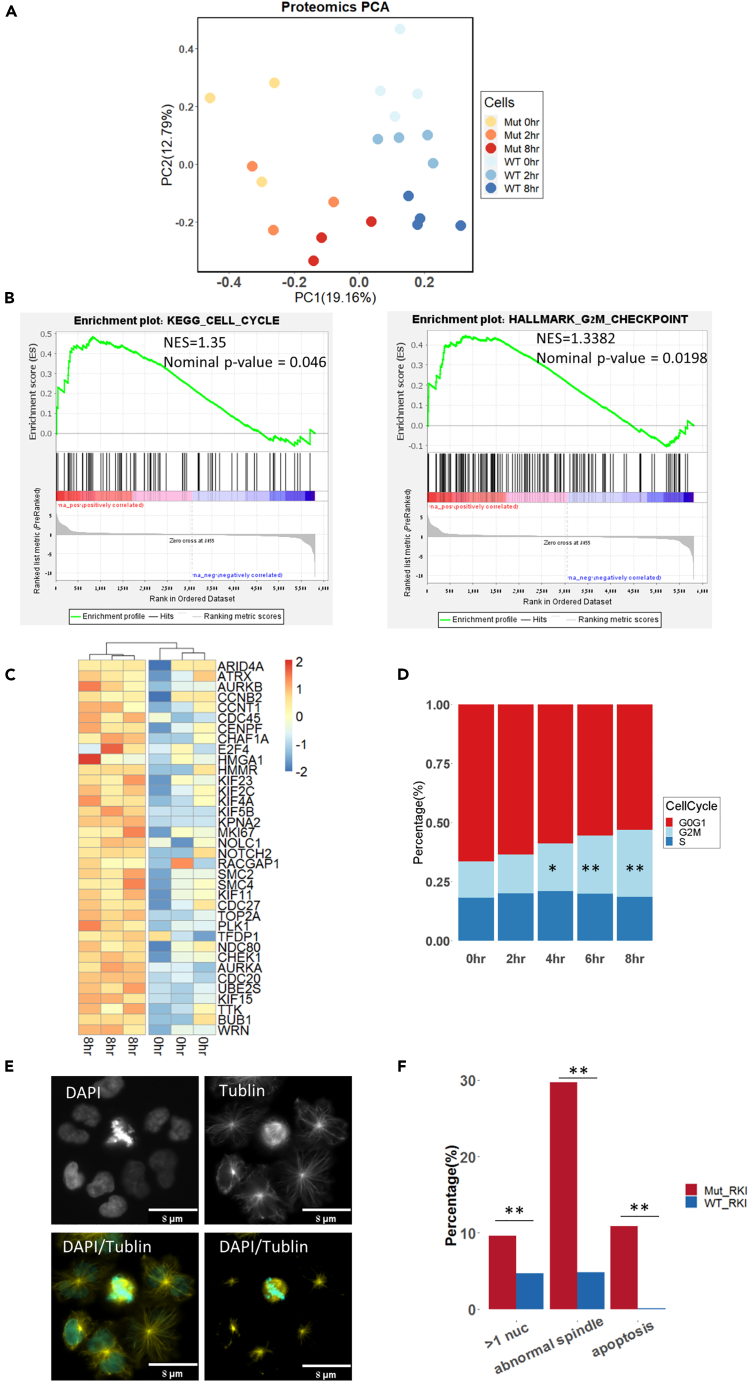
RKI-1447-driven mitotic stress (A) Principal-component analysis plot of protein expression in MOLM14 *SRSF2* WT/Mut cells before (0 h), after two- and 8-h exposure to RKI-1447 (0.5 μM). (B) GSEA analysis of protein expression pre-ranked based on the Log2 fold change between 8 h after exposure to RKI-1447 (0.5 μM) and before on MOLM14 Mut cells. Significant enriched pathways from the GSEA analysis included the cell cycle (NES = 1.35, Nominal p value = 0.046) and G2M checkpoint pathways (NES = 1.3382, Nominal p value = 0.0198). (Left) KEGG, Kyoto Encyclopedia of Genes and Genomes. (Right) Hallmark gene sets. NES, normalized enrichment score. (C) Heatmap of core enrichment genes for gene set G2M checkpoint between MOLM14 *SRSF2* Mut after 8 h exposure to RKI-1447 (0.5 μM) and before. (D) Cell cycle of MOLM14 *SRSF2* Mut cells before and after 2, 4, 6, and 8 h exposure to RKI-1447 (0.5 μM). Different cell-cycle stages are indicated with different colors in the plot. Significant increased G2/M cell-cycle stage was observed after 4, 6, and 8 h exposure to RKI-1447. t test, ∗p < 0.05; ∗∗p < 0.005; ∗∗∗p < 0.0005. (E) Representative image of abnormal spindles in MOLM14 *SRSF2* Mut cells after 24 h exposure to RKI-1447 (0.5 μM). Cells were stained with DAPI and a-tubulin. (F) Percentage of cells with multi-nuclei, abnormal spindles, and apoptosis in MOLM14 *SRSF2* Mut and WT after 24 h exposure to RKI-1447 (0.5 μM). Pearson’s chi-squared, ∗p < 0.05; ∗∗p < 0.005; ∗∗∗p < 0.0005.

To study the molecular and cellular mechanisms underlying the response of *SRSF2* Mut to ROCK inhibition, we exposed MOLM14, OCI-AML2, and MARIMO isogenic lines to RKI-1447 and measured the proteomic and RNA-seq profiles before exposure to the drug and 2 or 8 h later. Unbiased analysis of the proteomic data demonstrated a clear separation of protein expression between Mut and WT cells at all time points for all of the cell lines (Figures 3A, S4H, and S4I). In MOLM14 we observed a time-dependent proteome expression change in both Mut and WT (Figure 3A). These results suggest that RKI-1447 has a clear effect on the proteome of the Mut and WT cells; however, their baseline differences were maintained. Furthermore, gene set enrichment analysis of the protein expression between *SRSF2* Mut before and after exposure to RKI-1447 identified cell cycle and G2M checkpoint pathways as significantly upregulated in MOLM14 cells 8 h after treatment (Figures 3B and S5, and Datas S7 and S10). The upregulation of mitosis-related genes in the MOLM14 *SRSF2* Mut after RKI-1447 (Figure 3C) is a sign of mitotic arrest, suggesting cells were having hard time to complete mitosis. In accordance with these findings, treatment with RKI-1447 resulted in a higher percentage of cells in G2/M in both MOLM14 and OCI-AML2 (Figures 3D and S5). These data suggest a cell-cycle progression defect related to RKI-1447. While the proteomic analysis revealed evidence of a cell-cycle defect after exposure to RKI-1447, our aim was to gain more information from the RNA-seq data. Previous work on targeting of AML with *SRSF2/SF3B1* suggested that one possible approach to target such cells is by inhibiting splicing to modulate splicing profiles.^6^^,^^22^ Aberrant splicing analysis in our isogenic lines demonstrated a burst of aberrant splicing alterations 2 h after treatment in both the mutant and WT MOLM14, AML2, and MARIMO cells (false discovery rate [FDR]<0.05, Δ|PSI|> 10%, Figure S6). After 8 h, the amount of aberrant splicing events went back to baseline. These data suggest that RKI-1447 can possibly perturb RNA splicing. Interestingly, principal-component analysis (PCA) of the RNA-seq expression data also demonstrated that 2 h after exposure to RKI-1447 the *SRSF2* Mut cells had a distinct gene expression in MOLM14 and OCI-AML2 cells, though less clear in MARIMO cells (Figure S6). Together, the splicing and expression analysis indicated an RKI-1447-related effect in OCI-AML2 and MOLM14 2 h after exposure to the drug. To further explore this effect, we studied the aberrant spliced genes (exons skipped/included) which overlapped between MOLM14 mutant and OCI-AML2 mutant after 2 h of exposure to RKI-1447 (FDR <0.05). In summary, both the proteomic analysis and RNA-seq and cell-cycle analysis pointed to an abnormal cell cycle after exposure to RKI-1447 in both MOLM14 and OCI-AML2 *SRSF2* Mut cells. In the next step we aimed to understand why RKI-1447 introduced such a defect.

It is known that Rho GTPases are important regulators of mitosis and cytokinesis in mammalian cells through their ability to modulate microtubule dynamics and actin-myosin cytoskeleton.^13^^,^^23^ Accordingly, we stained our *SRSF2* WT/Mut cell lines with anti-α-tubulin and DAPI. We observed significantly more abnormal multipolar mitotic spindles, multi-nuclei, and apoptosis in *SRSF2* Mut cells compared with WT after exposure to RKI-1447 (Figures 3E and 3F). We also performed annexin V/propidium iodide (PI) assays for WT and mutant cells after exposure to RKI-1447 or knockdown rock1/2 via siRNA, and mutant cells demonstrated an increase in apoptosis compared to WT (Figures S6G and S6H). These findings suggest that RKI-1447 induces abnormal mitosis correlated with abnormal microtubule organization, supporting our proteomic and cell-cycle observations. To further explore the nature of the differential effect of RKI-1447 on the WT and *SRSF2* Mut cells, a comprehensive 3-dimensional (3D) characterization of the cytoskeleton and the nucleus in these cells was conducted.

### Differential effects of RKI-1447 on nuclear deformation and cytoskeleton organization in *SRSF2* WT/Mut cells

**Figure 4:**
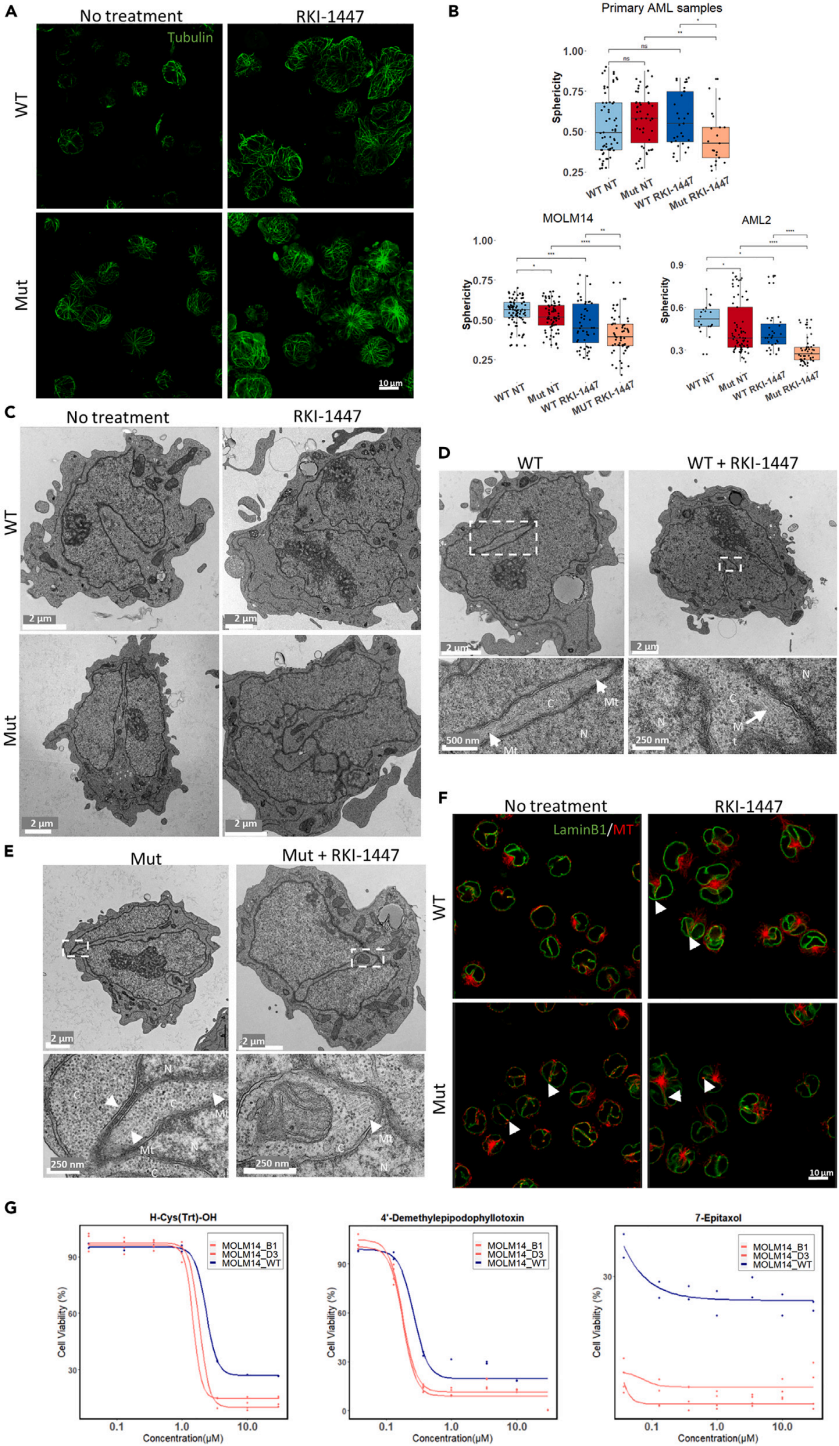
Confocal and TEM images of *SRSF2* WT/Mut cells before and after exposure to RKI-1447 *SRSF2* WT/Mut MOLM14 and OCI-AML2 cells were either left untreated or treated with 0.5 μM RKI-1447 for 24 h. One *SRSF2* Mut primary AML sample and one *SRSF2* WT primary AML sample were either left untreated or treated with RKI-1447 (1 μM) for 48 h before fixation. (A) Representative images of microtubules (shown in green), in *SRSF2* WT/Mut MOLM14 cells, untreated or treated with RKI-1447 (0.5 μM), were acquired using the Leica SP8 scanning confocal microscope. Cells were labeled with anti-α-tubulin antibodies. A series of confocal slices depicting the overall morphology of the microtubular network in these cells is shown in Figure S8. (B) 3D confocal images of DAPI-labeled nuclei were subjected to rendering and morphometric quantifications. Morphometric characteristics of rendered sphericity of primary AML samples (upper), MOLM14 cells, and AML2 cells (lower) are presented in box and whiskers plot format. Statistical analysis was performed using two-sided t tests; ∗p < 0.05; ∗∗p < 0.005; ∗∗∗p < 0.0005. (C) TEM showing a common presence of a deep (“half-way”) nuclear indentation (top-left image) in MOLM14 WT cells; In mutant MOLM14 cells, these nuclear indentations were considerably deeper, usually contacting the opposite side of the nucleus, thereby segmenting the nucleus (bottom-left image); treatment of WT cells with RKI-1447 induced conspicuous nuclear segmentation (top-right image). Note that segments are often connected to each other by extended sheets consisting of two nuclear membranes, inter-connected by the associated nuclear laminae; “hyper-segmentation” of the nuclei of the mutant cells (bottom-right). For higher magnification view, see also Figure S12. (D and E) TEM images illustrate the overall morphology of *SRSF2* WT (D) and mutant (E) MOLM14 cells, untreated or treated with RKI-1447 (0.5 μM) are shown in lower magnification (top panel) and higher magnification (bottom panel, corresponding to the white rectangles in the top panel). Nuclear regions and cytoplasmic regions are marked C and N, respectively. Arrows marked by “Mt” point to microtubules that are associated with the edges of the cytoplasmic insertions into the nucleus. The arrow in the “Mut” (bottom panel) points to the “interlobular sheet” consisting of the two nuclear membranes and the nuclear lamina running between them. (F) Cells were labeled with anti-LaminB1 antibodies to outline nuclear membrane (shown in green) and anti-α-tubulin antibodies to label microtubules (shown in red). 3D volumes were acquired on the Olympus confocal microscope. Representative confocal slices corresponding to the middle plane of the cells are shown. Note deep narrow folds of nuclear membrane that are especially prominent in mutant cells and upon RKI-1447 treatment. Red signal detected inside such invaginations points to the presence of microtubules (arrows). See also Video S1 and Figure S14. (G) Dose-response curves of three cytoskeleton modulators against *SRSF2* WT and Mut MOLM14 (B1, D3) cell lines. Viability of *SRSF2* WT and Mut MOLM14 cell lines was measured after 48 h exposure to three microtubule modifiers: H-Cys(Trt)-OH, 4′-Demethylepipodophyllotoxin and 7-Epitaxol at concentration of 0.037, 0.129, 0.369, 0.997, 3.49, 9.97, and 29.90 μM (see also Figure S17 for other MT modulators). The highest three concentrations were compared between WT and mutated clones by two-way ANOVA, followed by Dunnett’s *post hoc* test, see also Data S10.

As the cytoskeleton is one of the major regulators to cell and nuclear shape,^15^ we have compared the cytoskeletal (mostly tubulin and F-actin) in *SRSF2* WT/Mut MOLM14 and AML2 cells. Microscopy-based examination indicated some noticeable differences between WT and *SRSF2* Mut cells, manifested by a considerably larger projected area in the mutant cells compared to WT (Figure S7). Microtubule organization in the WT MOLM14 cells was mostly perinuclear, while, in the mutant cells, they were spread out radially from a central microtubule organizing center to the cell periphery (Figures 4A and S8). Exposure of the cells to RKI-1447 considerably enhanced the spreading of the cells, and the prominence of the microtubular network in both the WT and mutant cells.

Given the known involvement of microtubules in nuclear morphogenesis, we next examined the effects of the mutation and RKI-1447 treatment on the gross shape of the nuclei of DAPI-labeled cells using 3D light microscopy, in MOLM14, AML2 and primary AML cells. In MOLM14 WT cells the DAPI-labeled nuclei showed some heterogeneity, ranging from round to cashew-nut morphology (Figure S9). Mutant MOLM14 cells displayed higher level of heterogeneity, with larger nuclei, displaying multiple indentations (Figure S9). Rendering the WT and mutant nuclei, followed by quantitative morphometric analysis, indicated that the untreated mutant cells displayed more heterogeneous and significantly larger mean surface area and volume (Figure S9, Data S10) than the untreated WT cells. Upon treatment with RKI-1447 of MOLM14 cells, major changes in the overall shape of the nucleus occurred, manifested by apparent nuclear lobulation. Treatment of the mutant cells with RKI-1447 increased the nuclear indentations and induced extensive nuclear lobulation. To quantify the deviation of nuclei in MOLM14, OCI-AML2, and *SRSF2* Mut/WT primary AML cells (WT and mutant, untreated and treated with RKI-1447), we further calculated the nucleus sphericity. Indeed, the sphericity of MOLM14 *SRSF2* Mut cells significantly decreased after RKI-1447 treatment (Figure 4B, Data S10), in line with Figure S9. Consistent with our findings in MOLM14, both OCI-AML2 and primary *SRSF2* mut AML cells demonstrated significant lower sphericity value after RKI-1447 treatment, compared to *SRSF2* WT cells (Figures 4B, S10, and S11), while *SRSF2* WT primary AML cells did not similarly respond to RKI-1447 treatment, based on the sphericity analysis.

While the overall nuclear deformation, associating with the *SRSF2* mutation and RKI treatment, shown earlier, is compelling, the inherent capacity of 3D light microscopy to resolve fine intra-nuclear details is limited. To further explore the nature of the structural phenotype associated with the *SRSF2* mutations after RKI-1447 at higher resolution, we subjected the WT and mutant MOLM14 and AML2 cells (RKI-1447-treated and untreated) to comprehensive transmission electron microscopy (TEM) imaging (Figures 4C, S12, and S13). MOLM14 and AML2 WT cells displayed nuclei with variable overall morphology, commonly displaying bent morphology, with occasional broad indentations, shown here in longitudinal section (Figures 4C, S12, and S13). Consistent with the rendered light microscopy data, treatment of WT cells with RKI-1477 resulted in increased nuclear indentations. Untreated *SRSF2* mutant cells displayed deep and rather narrow (typically 200–300 nm) invaginations. Interestingly, essentially all the indentations, sectioned parallel to their long axis (WT and Mut, with or without RKI-1447), displayed a continuous cytoplasmic intrusion, from their tip to the nuclear periphery, suggesting that the indentations are rather narrow, yet wide, rather than cylindrical. This notion is further supported by cross section of the intrusions (see, e.g., Figures 4D [WT] and 4E [Mut+RKI-1447]). Moreover, we frequently noticed cytoplasmic intrusions that “went all the way,” effectively producing extended regions in which nuclear membrane areas (those of the intrusion and those of the other side of the nucleus) apparently interact with each other via the associated nuclear laminae (Figures 4C and S12B). Addition of RKI-1447 to the mutant cells resulted in conspicuous hyper-segmentation and hyper-lobulation of the nucleus (Figures 4C, S12B, and S13D) in both OCI-AML2 and MOLM14, supporting the significantly reduced sphericity observed by the light microscopy 3D imaging.

Interestingly, in MOLM14 WT cells, higher-power magnification of the cytoplasmic intrusions into the nucleus (Figures 4D and S12A’) revealed arrays of microtubules in the proximity of the indented nuclear membrane. High-power examinations of the deep indentations in *SRSF2* Mut MOLM14 cells reveal a prominence of microtubules that are located at the tips of the cytoplasmic intrusions into the nucleus and apparently “push on” the nuclear membrane at the protruding tip (Figures 4E and S12B’). Section of broad cytoplasmic intrusions that are prominent in RKI-1447-treated *SRSF2* Mut cells in both MOLM14 and AML2 cells reveals extended areas where the nucleoplasm was essentially dissected by the “invading” microtubule-rich cytoplasm (Figures 4E, S12B’, and S1D’).

The TEM data provided high-resolution visualization of the nuclear indentations and segmentation yet could not provide comprehensive 3D view of the nucleus-cytoplasm interplay and topology in these cells. To achieve such information, we double-immunolabeled *SRSF2* WT/Mut MOLM14 and OCI-AML2 cells and primary AML cells before and after exposure to RKI-1447 with tubulin and laminB1, which delineate the nuclear membrane, and visualized the cells by 3D confocal light microscopy.

### Abnormal microtubule system and sensitivity to microtubule modifiers of *SRSF2* Mut cells

The LaminB1 and tubulin staining reinforced the TEM data, directly demonstrating that the *SRSF2* mutations and RKI-1447 treatment strongly enhance the indentation of the nuclear membrane. This effect was observed in both MOLM14, AML2 cells (Figures 4F, S10, and S14, Video S1) and *SRSF2* Mut primary AML cells (Figures S15 and S16) after they were exposed to RKI-1447. The increased indentation and lobulation were associated with modulation the microtubule network, commonly manifested by the presence of microtubules in the laminB1-labeled indentations.

Video S1. Nucleus-cytoplasm interplay and topology of SRSF2 WT/Mut cells before and after exposure to RKI-1447, related to Figure 4F *SRSF2* WT and Mut MOLM14 cells, as indicated, were either left untreated or treated with 0.5 μM RKI-1447 for 24 h before fixation. Cells were labeled with anti-LaminB1 antibodies to outline nuclear membrane (shown in green) and anti-α-tubulin antibodies to label microtubules (shown in red). 3D volumes were acquired on Olympus scanning confocal microscope. The slices were acquired as described in STAR★Methods with a z-step size of 0.3 μm and made into .mp4 movies starting at the bottom, and proceeding toward the top.

Inspired by these imaging studies, we hypothesize that *SRSF2* Mut cells might also be vulnerable to treatment with microtubule modifiers, which could disturb the delicate interplay between the microtubule system and the nuclear membrane. We tested 34 cytoskeletal modifying compounds (including one ROCK inhibitor as positive control), which can bind and/or affect key cytoskeletal systems, such as tubulin, myosin, or actin. Among those we identified thirteen compounds, which were significantly more toxic to *SRSF2* Mut versus WT and/or showed clear dose response effect (Figures 4G and S17 and Data S10). Three compounds which significantly displayed differential dose effect on the *SRSF2* Mut cells were microtubule modulators 4′-Demethylepipodophyllotoxin (4'-DEMP) (a potent inhibitor of microtubule assembly),^24^ H-Cys(Trt)-OH (a specific inhibitor of Eg5 that inhibits Eg5-driven microtubule sliding velocity in a reversible fashion),^25^ and 2-Methoxyestradiol (2-MeOE2) (an inhibitor of polymerization of tubulin).^26^ Other compounds regulating the MT system, such as 7-Epitaxol (Figure 4G), Paclitaxel, Patupilone, Cabazitaxel, Epothilone A, and Docetaxel (Figure S17), demonstrated statistically significant effect but did not show a clear dose response, implying that the mutated cells were much more sensitive to the compound than the WT at concentrations below our screening range. These results further support the role of the MT system in the response of *SRSF2* Mut cells to RKI-1447.

### Nuclear F-Actin levels are increased in *SRSF2* Mut and modified by RKI-1447

In view of the extensive crosstalk between the tubulin-based and actin-based cytoskeletal networks, and the involvement of ROCKs in actin-myosin II-based cell contractility, we have labeled WT and mutant cells before and after exposure to RKI-1447 with DAPI and phalloidin. An increase of nuclear F-actin was observed in *SRSF2* Mut cells, compared to *SRSF2* WT at baseline and without treatment (Figure S7A). Following addition to RKI-1447, the ratio of nuclear actin/cytoplasm actin was increased from 0.52 to 0.68 in WT cells, while it was decreased from 0.86 to 0.67 in *SRSF2* Mut cells (Figure S7B). These findings expose yet another cytoskeleton system which is different between *SRSF2* Mut and WT cells.

Taken together, the discovery of *SRSF2* Mut cells sensitivity to RKI-1447 exposed cytoskeleton phenotypes of the *SRSF2* Mut cells. These phenotypes (abnormal cytoskeleton organization of both microtubules and F-Actin) are augmented by RKI-1447 treatment and thus could possibly explain why *SRSF2* Mut cells are sensitive to RKI-1447.

## Discussion

In the current study, we aimed at designing new models for HTDS against *SRSF2* Mut cells (Figure 1) with the aim of identifying safe and efficacious targeted therapies against *SRSF2* mutations (Figure 2). Although *SRSF2* mutations have generally been assumed to cause disease by inducing specific splicing defects, here we provide evidence that they are more sensitive to cytoskeletal and nuclear morphological changes. We discovered that RKI-1447, Rho-associated coiled-coil containing protein kinase inhibitor, significantly inhibited *SRSF2* Mut cells lines *in vitro* and also targeted primary human AML cells carrying *SRSF2* mutations in a PDX model (Figure 2). Furthermore, our investigations exposed a baseline abnormal nuclear/cytoplasm morphology among *SRSF2* Mut cells which are aggravated after exposure to RKI-1447. The severe deformed nuclear morphology and spindle irregularity were associated with abnormal MT organization, which are all enhanced by RKI-1447, and can lead to defective and deregulated cell cycle eventually leading to differential cell death (Figures 3 and 4). We also discovered that other MT modifiers (such as Taxol) can differentially target *SRSF2* Mut cells *in vitro* (Figure 4). We propose that the results presented here can help not just in identifying new compounds for targeting the pre-leukemic *SRSF2* Mut cells but also in understanding the mechanisms underlying *SRSF2* mutations.

Our study has both clinical and molecular-cellular mechanistic implications. From the clinical point of view, nearly all approaches to date to target *SRSF2* Mut HSPCs focus on splicing inhibition in myeloid malignancies based on the mechanism that *SRSF2* mutations cause defective splicing. However, such an approach has yet to show clear clinical benefits. In our study, we identified a different approach to target *SRSF2* Mut cells and validated it with primary human AML samples *in vivo*. Our comprehensive screening approach, using diverse cell lines, was fruitful as it enabled us to focus on ROCK inhibitors and MT modifiers as potential *SRSF2* Mut differential effectors. From the mechanistic point of view, we discovered that the selected ROCKi, RKI-1447, causes mitotic arrest and abnormal spindles in *SRSF2* Mut cells (Figure 3). In order to understand why *SRSF2* Mut cells were more sensitive to RKI-1447-induced mitotic stress, we extended our study to the cytoskeletal and nuclear morphology effects which are tightly regulated by the Rho-ROCK pathways.^27^ We performed quantitative analysis of nuclear morphology, which revealed significantly low sphericity of *SRSF2* Mut cells compared to that of untreated WT MOLM14 and OCI-AML2 cells, and the severity of this phenotype was extensively augmented by RKI-1447 treatment in MOLM14, OCI-AML2, and primary AML samples (Figure 4B). To gain deeper understanding of the role of specific cytoskeletal networks in nuclear deformation, and the fine organization of the affected interphase nuclei, we used TEM, which revealed cytoplasmic intrusions which segmented the nuclei and often induced the formation of two nucleus lobes that are connected with each other through a thin sheet, consisting of two nuclear membranes connected to each other via the nuclear laminae. These structures were prominently detected in *SRSF2*-mutated cells; this phenotype is reminiscent of the segmented pseudo Pelger-Huët anomaly (PHA) nuclei.^28^^,^^29^ The PHA is a blood laminopathy associated with mutations in the lamin B receptor (LBR) gene,^30^ and the characteristic of it is bi-lobed nuclei of leukocytes with symmetrical “dumbbell” or “pince-nez” appearance that is connected by a thin filament of chromatin.^31^ Acquired pseudo-PHA can be in MPN, MDS, and AML and can be induced by infections and medications.^32^ Our TEM results also revealed enrichment of microtubule bundles located at the tip and along the cytoplasmic intrusions, suggesting that they take an active mechanical role in the nuclear segmentation process (Figures 4D and 4E). This notion was further supported and substantiated by 3D light microscopy of the cells, following LMNB1 and tubulin immunostaining (Figures 4A and 4F, Video S1). ROCKs tightly regulated the organization of the actin system, and, toward that end, we stained F-actin sub-cellular distribution^33^ and found increased nuclear F-actin in *SRSF2* Mut cells, which was shown previously to be correlated with DNA damage.^34^ Previous studies have suggested that cells with *SRSF2* Mut display cell-cycle abnormalities, and that they might be sensitive to cell-cycle modulation due to DNA replication stress and R-loop formation.^12^^,^^35^^,^^36^

### Limitations of the study

Our study demonstrated that microtubule network is more prominent in *SRSF2* Mut, especially after RKI-1447 treatment. While our model system exposed sensitivity to RKI-1447 *in vitro* and *in vivo*, not all the cell lines we tested were equally sensitive to all ROCKis. This could be attributed to different genetic backgrounds and resistance to ROCKi in WT cells. To overcome this problem, we tested RKI-1447 on iPSC cells. While our findings expose abnormal nuclear shape and hyperactive microtubules associated with *SRSF2* mutations, it remains elusive why *SRSF2* mutations and possibly other splicing mutations modify the nucleoskeleton and cytoskeleton, at the molecular level. Furthermore, the identified targets may not necessarily be the direct contributors to the synthetic lethality observed in *SRSF2*-mutated cells. It remains unclear which exact signaling pathway is perturbed by RKI-1447, as ROCK has pleiotropic effects on cytoskeleton and cell growth, or potential off-targets might play a role in the underlying mechanism. Based on our results, we suggest the need for further investigations on the role of the ROCK pathways and nucleoskeleton and cytoskeleton system in MDS and AML with dysplastic changes. In this regard, a study with siRNA screening in AML samples identified ROCK as a target in AML with efficacy in 20% of primary AML samples.^37^ Our results suggest that modulating the cytoskeleton by RKI-1447 and possibly other microtubule modifiers can take advantage of the abnormal cytoskeleton induced by *SRSF2* Mut and open new ways to treat AML/MDS and pre-leukemia.

## STAR★Methods

### Key resources table

**Table:** 

REAGENT or RESOURCE	SOURCE	IDENTIFIER
**Antibodies**		

Anti-human CD45-BV510	BioLegend	HI30
Anti-human CD33-APC	BD Biosciences	WM53
Anti-human CD34-APC Cy7	BioLegend	581
Anti-human CD19-PE	BD Biosciences	HIB19
Anti-human a-tubulin	Sigma	Clone DM1A
Anti-human Lamin B1	Sigma	Clone 6K22
Rabbit anti-Rock1	Cell Signaling Technology	C8F7
Rabbit anti-Rock2	Cell Signaling Technology	E5T5P

**Critical commercial assays**		

Human CD45 depletion Kit II	EasySep	18259
CellTiter-Glo	Promega	G7572
Human CD3 positive selection kit	EasySep	17851

**Deposited data**		

Raw data	This paper	NIH Sequence Read Archive (SRA) accession numbers PRJNA804684

**Oligonucleotides**		

sgRNA for generating *SRSF2* mutated cell lines: GCGGCTGTGGTGTGAGTCCG	This paper	N/A
ssODN for generating *SRSF2* mutated cell lines: GGGGCCGTGCTGGACGGCCGCGAGCTGCGGGTGC AAATGGCGCGCTACGGCCGCCACCCGGACTCACAC CACAGCCGCCGGGGACCGCCACCCCGCAGGTACG GGGGCGGTGGCTACGGA	This paper	N/A
PCR primers for SRSF2 region: Fwd: CTACACGACGCTCTTCCGATCTctcagccccgtttacctg, Rev: CAGACGTGTGCTCTTCCGATCTctgaggacgctatggatg	This paper	N/A

### Resource availability

#### Lead contact

Further information and requests for resources and reagents should be directed to and will be fulfilled by the lead contact, Liran Shlush (liran.shlush@weizmann.ac.il).

#### Materials availability

This study did not generate new unique reagents.

#### Data and code availability

• Data: All data needed to evaluate the conclusions in the paper are present in the paper and/or the Supplementary Materials or from the corresponding author upon reasonable request. RNA-seq and proteomics data are publicly available through NIH Sequence Read Archive (SRA) accession numbers PRJNA804684.
• Code: Not applicable
• Any additional information required to reanalyze the data reported in this work paper is available from the lead contact upon request.

### Experimental model and study participant details

#### Study design

The objective of this study is to identity safe and efficacious targeted therapies against *SRSF2* mutations in hematopoietic system and understand the mechanisms behind. For all experiments, the number of replicates, statistical test used, and P values are reported in the figure legends. For human samples, primary peripheral blood samples were obtained with informed consent from AML patients through the Leukemia Tissue Bank at Princess Margaret Cancer Centre in accordance with regulated procedures approved by the Research Ethics Board of the University Health Network (REB 01-0573-C). All patients provided written informed consent for the usage of their samples for research purposes and for the usage of their clinical and biological data. For murine studies, mice were derived germ-free and housed at the Weizmann Institute of Science animal facility under institutional animal care and use committee approval (11790319-2). Cages of mice were randomly assigned to different treatment groups. Full experimental details can be found in the Supplementary Materials and Methods.

### Method details

#### Cell lines

K562 cell line was purchased from the Stem Cell & Advanced Cell Technology Unit at the Weizmann Institute of Science and *SRSF2* Mut K562 cells was a generous gift from Omar Adel-Wahab’s lab; OCI-AML2, MOLM-14 and MARIMO cell lines were kindly donated by the Deininger/O'Hare Lab at the Huntsman Cancer Institute, University of Utah. All cells were cultured in RPMI-1640, 10% FBS and 1% P/S (01-100-1A, 04-007-1A and 03-031-1B, respectively, Biological Industries).

#### Design of CRISPR guides and ssODN

All oligo sequences were designed using Benchling Life Sciences R&D Cloud (https://benchling.com/). A 20bp sgRNA (GCGGCTGTGGTGTGAGTCCG) was designed for a SpCas9 (3’ side, PAM=NGG) system. The guide was designed to cut the *SRSF2* exon 2, five bases downstream of the P95 SNP. A 120bp single-strand donor oligonucleotide (ssODN) was designed with as single mismatch so that it both altered the PAM motif as well as encoded for histidine instead of proline (GGGGCCGTGCTGGACGGCCGCGAGCTGCGGGTGCAAATGGCGCGCTACGGCCGCCACCCGGACTCACACCACAGCCGCCGGGGACCGCCACCCCGCAGGTACGGGGGCGGTGGCTACGGA). Mutations in the *SRSF2* hotspot region around P95 were generated. All the CFD score of predicted off-target loci are low (below 0.5), except for an intergenic region between the genes CT45A6/CT45A5-SAGE1 (Data S1).

#### Cell transfection

All cells were transfected in 20μl 16-well strips using a Lonza 4D-NucleofectorTM. Cells were sub-cultured 48hr prior to transfection at a concentration of 300,000 cells/ml. OCI-AML2 cells were transfected in SF solution (V4XC-2032, Lonza), 300,000 cells/rxn, using DN-100 program. MOLM-14 cells were transfected in SF solution, 1,000,000 cells/rxn, using DP-115 program. MARIMO cells were transfected in SF solution, 500,000 cells/rxn, using DN-100 program. K562 cells were transfected in SF solution, 200,000 cells/rxn, using FF-120 program. The IDT Alt-R® CRISPR-Cas9 System Delivery of ribonucleoprotein complexes in HEK-293 protocol (v3.1) was used for reagent ratios. In brief, 2.1μl PBS (02-023-1A, Biological Industries), 1.2μl Alt-R® CRISPR-Cas9 sgRNA (100μM, IDT), 1.7μl Alt-R® S.p. Cas9 Nuclease V3 (61μM, IDT), for a total of 5μl/rxn. 1 μl Alt-R™ HDR Donor Oligo (200 μM, IDT) was added to each reaction. Following transfection cells were washed with medium, divided to two wells and cultured. Four days following transfection, bulk cells from one of the duplicate wells was centrifuged and DNA was extracted by way of lysis. 80μL of 50mM NaOH was added to each cell pellet, and heated at 99°C for 10 min. Cell lysate was then cooled on ice and 8μL of 1M Tris pH 8.0 was added.

#### Next generation sequencing library preparation

Libraries were prepared according to previously described STAR Methods.^38^ In brief, primers for the amplification of *SRSF2* P95 were designed, and 5’ adaptors were added to their sequence: (Fwd: CTACACGACGCTCTTCCGATCTctcagccccgtttacctg, Rev: CAGACGTGTGCTCTTCCGATCTctgaggacgctatggatg). Each PCR reaction contained 5μL of NEBNext® Ultra™ II Q5® Master Mix (M0544S, NEB), 0.5μL of each above primer (10μM), 4μL of cell lysate. The reaction was placed in an Eppendorf Mastercycler pro Thermal Cycler and the following protocol was initialized: 98°C for 30 sec; 33 cycles of 98°C for 10 sec and 65°C for 30 sec; 65°C for 5 min. The product of this reaction (‘PCR1’) was diluted 1:1000 and served as a template for the following reaction. Next, dual sequencing barcode were ordered according to the following formation: Fwd primer:

AATGATACGGCGACCACCGAGATCTACAC[Fw_Index_D5XX]ACACTCTTTCCCTACACGACGCTCTTCCG; Rev primer: CAAGCAGAAGACGGCATACGAGAT[Rev_Index_D7XX]GTGACTGGAGTTCAGACGTGTGCTCTTCCG. The second PCR reaction (‘PCR2’) contained 2.5μL of NEBNext® Ultra™ II Q5® Master Mix, 0.5μL nuclease-free water, 1μL of the diluted PCR1 template, and 1μL of the above barcode mix (2.5 μM). A total of 5μL were placed in the thermal cycler using the same protocol as above, for 28 cycles. The resulting PCR2 reaction was cleaned of any residual enzyme, nucleotides, and primer dimers traces, according to the recommended size selection protocol using AMPure XP SPRI magnetic beads (Beckman Coulter) at a volume ratio of x0.7.

#### Single cell-sorting

Four to seven days following the abovementioned transfection, bulk cells were stained with Propidium Iodide (556463, BD Pharmingen™) as per manufacturers’ instructions. Cells were sorted using a BD FACSAria™ III Cell Sorter, one cell per well, into Nunc™ Edge™ 96-Well Microplates (167425, Thermo Fisher). Cells were cultured for 2-4 weeks until colonies were visible, after which 100μL of cells and medium were transferred to Axygen® 96-well PCR plates (PCR-96-FS-C, Corning) and centrifuged at 400g for 5 minutes. The supernatant was decanted, and DNA was extracted from cell pellets according to the lysis protocol described above. The resulting DNA was prepared according to the library preparation protocol described above and proceeded to sequencing for genotyping of colonies.

#### Compound libraries

Three commercial libraries were used in the screening process: the Bioactive library (Selleck Collection, n=3727), the Kinom Set (n=187), and the Epigenetic chemical probe library (both from Structural Genomics Consortium, n=97). ID inconsistent compounds were removed (N=23).

#### Preparation of siRNA stock

ON-TARGETplus human ROCK1 siRNA (L-003536-00-0005 NM_005406) and ON-TARGETplus Human ROCK2 siRNA (L-004610-00-0005 NM_001321643 NM_004850) and ON-TARGEplus non-targetin Pool were purchased from Dharmacon. SiRNA(5nmol) were resuspended in 50 μl RNase-free water or siRNA suspension buffer to obtain a 100μM siRNA stock solution. Heat the tube to 90°C for 1 min Incubate at 37°C for 60 min and stock at -20°C.

#### siRNA transfection

Cells were sub-cultured 48hr prior to transfection at a concentration of 300,000 cells/ml. Cells were transfected in 20μl 16-well strips using a Lonza 4D-NucleofectorTM. Cells were transfected in SF solution, 500,000 cells/rxn, using DP-115 program. 250nM siRNA pool targeted ROCK1/ROCK2 and non-targeted siRNA pool added to different reaction. Following transfection cells were divided to two wells and cultured. 48 hours after transfection, the number of the viable cells are counted manually and normalized to non-targeted well.

#### Western blot

48 hours after siRNA treatment, cells were harvested by a PBS wash followed by incubation for 10 min with ice–cold RIPA lysis buffer (150 mM NaCl, 1% NP-40, 0.1% SDS, 0.5% sodium deoxycholate, 50 mM Tris pH 7.4, and protease inhibitors 1:1,000 (Sigma, P8340)). The resulting lysate was quantified by Pierce Rapid Gold BSA protein assay kit (Thermo Scientific, A53225), and each sample was diluted to reach the same protein concentration. Each sample was resuspended in 1x Laemmli sample buffer (10% glycerol, 60 mM Tris-HCl pH 6.8, 1% (v/v), 2-mercaptoethanol, 0.02% bromophenol blue, 0.2% SDS) and denatured at 95C for 5 minutes. Equal amounts of protein lysates (20 μg) were analyzed by SDS-PAGE, and blotted to a nitrocellulose membrane (Pall Corporation, 66485) 1 h at 300 mA in transfer buffer (15 mM Tris-base, 120 mM glycine). Membranes were blocked in 5% (w/vol) milk powder in 0.05% Tween-20/TBS (TBS-T) and probed for 16 hours at 4°C with the indicated antibodies: rabbit anti-ROCK1 (C8F7) (Cell Signaling Technology, CST-4035S), rabbit anti-Rock2 (E5T5P) (Cell Signaling Technology, CST-47012S), and rabbit anti-Gapdh (D16H11) (Cell Signaling Technology, #5174). Subsequently, membranes were incubated for two hours at room temperature with Rabbit (Invitrogen, A16104) horseradish peroxidase-conjugated anti-IgG secondary antibodies diluted 1:5,000, and bands visualized with ECL reagent (100 mM Tris-HCl pH 8.6, 1.25 mM luminol, 200μM coumaric acid, and 0.01% H2O2) on a Biorad ChemiDoc XRS+ and quantified using (Fiji) ImageJ software. Each band was individually selected and circumscribed with an ROI of equal size, followed by measurement of the peak area of the obtained histograms. Rock1 and Rock2 intensities were normalized to Gapdh.

#### RNA extraction

RNA was extracted from MARIMO A2, A10, C1, MOLM14 B1, D3, D5 and AML2 B7, B3, F6, E9 before and after two and eight hours of exposure to RKI-1447 (0.5μM). RNA of all the cell lines was extracted with Qiuck-RNA MagBead (Zymo, R2132) according to the manufacturer’s instructions. Raw data of RNA-seq was uploaded to NCBI (Accession number PRJNA804684).

#### Library preparation for bulk RNA-seq

Sequencing Libraries were prepared using INCPM mRNA Seq. NovaSeq 200 cycles reads were sequenced on 2 lane(s) of an Illumina novaseq. The output was ∼54 million reads per sample.

#### Gene expression analysis

Gene expression pipeline was run with UTAP^39^ as following: Reads were trimmed using cutadapt (parameters: -a ADAPTER1 -a “A{10}” -a “T{10}” -A “A{10}” -A “T{10}” –times 2 -q 20 -m 25). Reads were mapped to genome hg19 using STAR v2.4.2a (parameters: –alignEndsType EndToEnd, –outFilterMismatchNoverLmax 0.05, –twopassMode Basic). The pipeline quantifies the Genecode annotated genes: hg19-genecode.genes.gtf. The annotation version and date are description: evidence-based annotation of the human genome (GRCh37), version 19 (Ensembl 74). Counting was done using STAR. Further analysis is done for genes having minimum 5 read in at least one sample. Normalization of the counts was done using DESeq2 with the betaPrior set to True. Raw P values were adjusted for multiple testing using the procedure of Benjamini and Hochberg.

#### Cell viability assay

Compounds were dispensed in 384-well plates using an ECHO® 555 liquid handler (Labcyte) and sealed. On day of experiment, the concentrations of isogenic and respective wildtype cell lines were counted using a Countess™ II FL (InvtrogenTM) and re-suspended at 40,000 cell/ml. 50μL of medium was dispensed using a Multidrop™ Combi Reagent Dispenser (Thermo Fisher), bringing the total number of cells in each well to 2000. Cells were incubated for 48 hours. On day of measurement, plates were centrifuged, and supernatant was removed using a Washer/Dispenser II (GNF Systems). A Washer/Dispenser II was then used to dispense CellTiter-Glo® (G7572, Promega) as per the manufacturer’s instructions. The luminescence signal was then measured using a PHERAstar® FSX (BMG Labtech) and results were analyzed using Genedata Screener®. The viability of treated cells was normalized to a vehicle control, contained on each plate. The highest three concentrations were compared between WT and Mutated clones by two-way ANOVA, followed by Dunnett’s post-hoc test for Figures 1E and 1G.

#### *In vivo* assay

NSG mice (n=5-10/sample) were injected with half million to five million CD3 depleted AML cells from the peripheral blood of patients with *SRSF2* mutated AML (#209945 and #830163) and patients without *SRSF2* mutated AML (#150279, #150316 and #150664) intrafemorally. One of the *SRSF2* mutated AML sample (#800667) was injected into SGM3 mice with the same method. NSG mice (n=5-10/sample) were injected with 80,000 to 150,000 CD34^+^ cells from three mobilized peripheral blood samples (#141451, # 141509 and #141494) intrafemorally. On day 35 the animals were randomized to RKI-1447 or a carrier control. RKI-1447 was administered i.p. at a dose of 50mg/kg daily for 21 days. On day 56 mice were sacrificed and analyzed for human engraftment by measuring the percentage of human CD45^+^ cells with flow cytometry. Antibodies against the flowing molecules were used: CD45-BV510 (HI30, BioLegend, 1:200), CD33-APC (WM53, BD Biosciences, 1:100), CD34-APC Cy7 (BioLegend, 581, 1:100), CD15-BV421 (BioLegend, W6D3, 1:100), CD38-PE Cy7 (BioLegend, HIT2,1:100), CD3-FITC (BD Biosciences, UCHT1,1:100), CD19-PE (BD Biosciences, HIB19, 1:200), PI (BD Biosciences, 556463,1:100). The percentage of CD19^+^, CD33^+^ and CD19-CD33- human CD45^+^ (hCD45) cells in engrafted murine bone marrow were measured with by flow cytometry to determine myeloid or multi-lineage engraftment. The presence of *SRSF2* Mutations of the CD45^+^ human engrafted cells are sequenced with target sequence of P95 region. Library preparation and sequencing are performed as we mentioned at the previous section. The weight of NSG mice was measured before and after drug administration, no significant change of weight or death was observed between control group and experiment group (data not shown).

#### DNA extraction of the grafts

The human CD45^+^ grafts of NSG mice are separated with EasySep™ Human CD45 Depletion Kit II (Catalog 18259) for each mouse and then DNA of the cells are extracted immediately with QIAamp® DNA Micro kit (Qiagen 157032361) according to the manual instruction.

#### Cell lines for hepatoxicity

THLE-2 (CRL-2706™) were purchased from ATCC and were grown in the BEGM Bullet Kit (CC-3170) from Lonza. Besides the additives contained in the kit, the medium was further supplemented with 5 ng/mL EGF (Sigma), 70 ng/mL phosphoethanolamine (Sigma) and 10% FBS. The plates for the THLE-2 needed to be pre-coated with a mixture of 0.01mg/mL fibronectin, 0.05mg/mL of PureCol™ EZ Gel Solution (Sigma) and 0.01mg/mL of BSA dissolved in BEBM medium (Lonza). The coating medium was aspirated before seeding.

#### Assessment of compound toxicity in THLE-2

THLE-2 cells were exposed to the compounds in a 9-point, 2-fold dilution dose response series with a 100μM as upper limit, for 72 hours. Following the 72 hours of exposure to the test compounds, cell viability was determined by measuring the concentration of cellular ATP (CellTiter Glo, Promega). The luminescence signal was measured on a Pherastar FS multi-mode plate reader (BMG Labtech). Each data point was run in triplicate.

#### The predictor™ hERG assay

The assay was performed in accordance to the manufacture protocol (Invitrogen, #PV5365). Shortly, a small volume medium binding 384-well plates (Greiner, #784076), were preplated with RKI1447, Cisapride (positive control), and DMSO (vehicle) using an ECHO® 555 liquid handler (Labcyte). Additional controls – E-4031 (kit positive control), blank, free tracer and negative control were added in accordance to the manufacture protocol. The FP signal was measured using 540/590 fluorescent polarization filter of PHERAstar® FSX (BMG Labtech) and results were analyzed using Genedata Screener®. The polarization (mP) signal was normalized to negative/positive controls.

#### Metabolic stability assay

2 mM Compounds in DMSO are prepared and stored at -20 degrees. Pooled Human Liver Microsomes (Mixed Gender Sigma M0317) are diluted to 0.6 mg/ml in 100 mM potassium phosphate buffer (pH 7.4) with 1.0 mM NADPH (Sigma N5130). Compounds (1 μM final) are incubated with microsomes and NADPH at 37 degrees for a time course using thin-walled PCR tubes and a thermocycler set to constant temperature. At each time-point, 5 μl aliquots are removed to 25 μl ice cold Acetonitrile containing 10 μg/ml Kaempherol (Sigma K0133) as an internal control. Centrifuge 2500xg for 15 minutes, add 20 μl ice-cold DDW and repeat centrifuge. Transfer to 384-well plate (Waters 186002631), seal with aluminum seal and store at -80 degrees. Compounds are quantified by LC/MS. Data is normalized to percent of input compound in buffer without microsomes and time-course is plotted with Graphpad Prism.

#### CD34^+^ cells separation for hematotoxicity

Enrichment of human CD34^+^ cells from PBMC of healthy individual was performed according to the manufacturer’s instructions (Miltenyi Biotech, Bergisch Gladbach, Germany). Seed CD34^+^ cells in 384-well plate at a density of 1000 cells per well in 70ul SFEMII medium with different cytokine cocktail for myeloid, erythroid and megakaryocytic lineages, culture in an incubator for 7 days at 37 degrees in a humidified atmosphere containing 5% CO2. Cytokines for myeloid differentiation: rh Flt-3 Ligand (20ng/ml, Z02926-50, GenScript), rh Stem Cell Factor (100ng/ml, Z02692-50, GenScript), rh IL-3 (10ng/ml, Z03156-50, GenScript), rh IL-6(50ng/ml, Z03034-50, GenScript), rh GM-CSF (20ng/ml, Z02983-50, GenScript); Cytokines for erythroid differentiation: rh Stem Cell Factor (100ng/ml, Z02692-50, GenScript), rh IL-3 (10ng/ml, Z03156-50, GenScript), Erythropoietin (3ng/ml, Z02975-50, GenScript); Cytokines for megakaryocytic differentiation: rh Stem Cell Factor (100ng/ml, Z02692-50, GenScript), rh IL-6(50ng/ml, Z03034-50, GenScript), rh IL-9(10ng/ml, Z03013-50, GenScript), rh Thrombopoietin (100ng/ml, 300-18-10ug, GenScript).

On Day 7, cells were treated with different concentrations of RKI-1447. On Day 9, the viability of cells is detected with CTG assay, and the duplicate plate was transferred to 96-well plate and stained with the antibodies for flow cytometry to determine the fraction of differentiated cells after treated with RKI-1447. Antibodies used for flow cytometry of hematotoxicity: CD42b-APC (303912, BioLegend,1:100) was used for megakaryocytic lineage; CD11b/MAC-1-APC-Cy7 (BD, PMG-557754, 1:100), CD66b- BV421 (BD, 562940, 1:100), CD15-BV650 (BD, 564232, 1:100) and CD14-PE-Cy7 (BD, 557742, 1:100) were used for myeloid lineage; CD71-FITC(BD, 347513, 1:100 ) and CD235a-PE (BioLegend, 349105, 1:100) were used for erythroid lineage; CD34-pecy5.5 (eBioscience, 46-0349-42, 1:100) was used for stem/progenitors; PI (BD Biosciences, 556463,1:100) was used to exclude dead cells.

#### iPSCs

*SRSF2* P95L and isogenic WT iPSCs were generated and differentiated into HSPCs as previously described.^9^^,^^21^ iPSC-derived HSPCs were plated at a density of 8k cells per well in 96-well plates and treated with RKI-1447 (3μM) or DMSO for 48 hours. Cell viability was measured for both in the vehicle and in RKI-1447 treated cells with CellTiter-Glo® (G7572, Promega) according to the manufacturer’s instructions. The cell viability of RKI-1447 treated cells were normalized to the vehicle treated cells in either WT or mutant. The statistic was done with Mann-Whitney test.

#### Sample preparation and data processing for LC/ MS

The samples were subjected to tryptic digestion using an S-trap. The resulting peptides were analyzed using nanoflow liquid chromatography (nanoAcquity) coupled to high resolution, high mass accuracy mass spectrometry (Fusion Lumos). Each sample was analyzed on the instrument separately in a random order in discovery mode. Raw data was processed with MaxQuant v1.6.6.0. The data was searched with the Andromeda search engine against the human proteome database appended with common lab protein contaminants and the following modifications: Carbamidomethylation of C as a fixed modification and oxidation of M and protein N-terminal acetylation as variable ones. The LFQ (Label-Free Quantification) intensities were calculated and used for further calculations using Perseus v1.6.2.3. Decoy hits were filtered out, as well as proteins that were identified on the basis of a modified peptide only. The common contaminates are labeled with a ‘+’ sign in the relevant column. The LFQ intensities were log transformed and only proteins that had at least 2 valid values in at least one experimental group were kept. The remaining missing values were imputed. Proteins labeled with a “+” sign are removed and then proceeded with principal component analysis in R with prcomp function. The gene names were ranked based on fold change of the proteomics between different conditions and analyzed using the GSEA software version 4.1.0. Number of permutations: 1000. Gene sets database h.hall.v7.4.symbols.gmt [Hallmarks] and c2.cp.kegg.v7.4.symbols.gmt [Curated] were used.

#### Phosphoproteomics

Cells were incubated with RKI-1447 for 0hour, 2hours and 8hours.The cells were pelleted and washed twice with PBS. The cells were lysed using 5% SDS in 50 mM Tris pH 7.6 for a final protein concentration of 2mg/ml. The lysates were snap frozen and kept at -80C. The cell lysates were sonicated in a sonication bath. The samples were subjected to tryptic digestion using an S-trap followed by an automated phospho enrichment using IMAC-Fe3+. The resulting peptides were analyzed using nanoflow liquid chromatography (nanoAcquity) coupled to high resolution, high mass accuracy mass spectrometry (Q Exactive HF). Each sample was analyzed on the instrument separately in a random order in discovery mode. Raw data was processed with MaxQuant v2.0.1.0. The data was searched with the Andromeda search engine against the human proteome database appended with common lab protein contaminants and the following modifications: Carbamidomethylation of C as a fixed modification, oxidation of M, protein N-terminal acetylation and phosphorylation of STY as variable ones. The phospho sites intensities were calculated and used for further calculations using Perseus v1.6.2.3. Decoy hits were filtered out, and the data were log2-transformed, followed by subtracting the median of each sample. Only sites that had at least 2 valid values in at least one experimental group were kept. The remaining missing values were imputed by a low constant (-7).

#### Cell cycle assay

Cells were collected and washed twice with PBS. Cells were suspended with 0.5 ml PBS and 4.5 ml of ice-cold EtOH 70% were added. Cells were mix well in the tube and kept on ice for at least 2 hours. Centrifuge the ethanol-suspended cells. Decant ethanol thoroughly. Resuspend the cell pellet in 5 ml PBS, and centrifuge. Resuspend cell pellet in 1 ml PI/Triton X-100 staining solution with RNase. Keep it either 15 min at 37°C or 30 min at room temperature. Set up and adjust the flow cytometer for excitation with blue light and detection of PI emission at red wavelengths. Measure cell fluorescence in the flow cytometer (Cyto Flex). Use the pulse width–pulse area signal to discriminate between G2 cells and cell doublets and gate out the latter. Read PI at linear scale. The analysis of cell cycle was performed with Flowjo with the Watson pragmatic model.

#### Propidium iodide (PI)/Triton X-100 staining solution with RNase A

To 10 ml of 0.1% (v/v) Triton X-100 (Sigma 93443-100ML) in PBS add 2 mg DNase-free RNaseA (Sigma R6513-50MG) and 200 μl of 1 mg/ml PI (Sigma P4170-25MG). Prepare freshly.

#### Immunofluorescent cell labeling

Suspension of MOLM14 and AML2 cells, at the density of 300,000 cells/ml were cultured for 24 hours either in fresh RPMI 10%FBS 1%P/S medium, or in medium containing 0.5 μM RKI-1447. Frozen primary *SRSF2* Mut/WT AML samples were thawed and CD3^+^ cells were depleted with CD3 positive selection kit (EasySep Human CD3 Positive Selection Kit II, STEMCELL Cat.17851). Primary AML cells were incubated for 72 hours either in fresh RPMI 10%FBS, 1%P/S medium supplemented with SCF (100ng/μl), IL3 (10ng/ng/μl), IL6 (20ng/μl), TPO (10ng/μl) or in medium containing 1μM RKI-1447.

The cells were then plated onto optical plastic bottom 24 well dishes (IBIDI, Gewerbehof Gräfelfing) or 96-well optical plate (Thermo Scientific Nunc, Cat.164588). The cells were allowed to adhere to the well for 10 minutes^40^ and then washed with PBS and fixed-permeabilized for 1 minute with cytoskeleton stabilizing buffer:10 mM MES, 150 mM NaCl, 5 mM EGTA, 5 mM MgCl2, and 5 mM glucose, pH 6.1 containing 0.5% glutaraldehyde (Electron Microscopy Science) and 0.25% TrytonX100, and postfixed with 1% glutaraldehyde for 10 minutes.^41^ The cells were then washed three times with PBS and treated for 10 minutes with 1mM sodium borohydride to reduce non-specific cellular auto fluorescence.

Cells were labeled with either mouse monoclonal antibodies against α-tubulin (clone DM1A dilution 1:500, Sigma) or with rabbit monoclonal antibodies against Lamin B1 (clone 6K22, dilution 1:100, Sigma). Secondary antibodies used in these experiments were Alexa-488 conjugated goat-anti mouse or anti-rabbit antibodies 1:200 dilution (Invitrogen) was used. Actin was labeled using TRITC- Phalloidin (Sigma) 5ug/ml; and nuclei were labeled with DAPI (Sigma). For Figure 3F, the apoptotic cells were counted manually based on the cell morphology, characterized by shrinkage or membrane blebbing, and the nuclei morphology, characterized by condensation or fragmentation, using DAPI staining, which typically reveals bright spots or irregular shapes.

#### Confocal microscopy and image analysis

The cells were imaged on two confocal microscopes, the first an Olympus confocal FluoView FV1000 IX81 confocal laser-scanning microscope equipped with a Diode laser with 405 nm emission, an Argon-ion laser with 488 nm emission and a Helium-Neon laser with 560 nm emission laser for dye excitation. Confocal microscopy scanning was sequential with a 4 μs/pixel dwell time. Emission signals were collected using PMTs. Images were sampled at 1600 by 1600 pixels with a bit depth of 12, using a 60x oil immersion objective (NA 1.35), and a 2X software zoom. The pinhole size used was 2 AU (119 μm). Z stacks were acquired with a step size of 0.3 μm. The images were visualized by Fluoview Olympus confocal software and afterwards processed by Imaris or ImageJ software.

A second set of confocal images were acquired, using an inverted Leica SP8 STED3X confocal microscope, equipped with internal Hybrid (HyD) detectors and Acusto-Optical Tunable Filter (Leica microsystems CMS GmbH, Germany) and The White light laser (WLL) excitation laser ranging from 470 - 670 nm. Dye excitation was performed using the white and diode laser. Emission signal was collected using an internal HyD detector. Imaging was acquired with STED-objective HCX PL APO 93x/1.30 GLYC STED White motCORR. The scanning was sequential and bi-directional with a scanning speed of 631 or 700 Hz. Images were sampled at 2328 by 2328 pixels or 2504 by 2504 with a bit depth of 16 and a pixel size of 0.042/0.042/0.35 μm or 0.039/0.039/0.286 for x/y/z dimensions. The zoom was set to 1.28, the line average 2 with a pinhole size of 0.89 AU (142.07 μm). Z stacks were acquired using the galvo stage, with a step size of 0.38 μm. The deconvolution of the Leica software was applied, and the acquired images were visualized using LASX software (Leica Application Suite XLeica microsystems CMS GmbH).

Three-dimensional reconstruction and image analysis were carried out using the Imaris software, Oxford instruments (Bitplane) package, version 9.7.1. For the 3D nuclear shape analysis, their surfaces were rendered by manual thresholding that was kept constant, and the data such as area and volume was exported to excel files for statistical analysis.

The actin analysis was performed using ImageJ (Fiji). First the cells were thresholded by actin (TRITC phalloidin staining), using the Otsu threshold, then a mask was created. This was repeated for DAPI, to mask the nucleus. The mask created by actin was eroded x 12 (pixels) as to exclude the cortical actin contribution. The masks were subtracted, as a new cytosolic mask was created. The intensity of actin was measure in the nucleus mask, and in the cytosolic mask and the ratio was calculated for all four samples.

#### Transmission electron microscopy

Cells were fixed for 24 hours with 4% paraformaldehyde, 2% glutaraldehyde in 0.1 M cacodylate buffer containing 5 mM CaCl2 (pH 7.4), postfixed in 1% osmium tetroxide supplemented with 0.5% potassium hexacyanoferrate trihydrate and potassium dichromate, stained with 2% uranyl acetate in double-distilled water for 1 hour, dehydrated in graded ethanol solutions and embedded in epoxy resin. Ultrathin sections (70 nm) were obtained with a Leica EMUC7 ultramicrotome and transferred to 200 mesh copper transmission electron microscopy grids (SPI). Grids were stained with lead citrate and examined with a Tecnai SPIRIT transmission electron microscope (Thermo Fisher Scientific). Digital electron micrographs were acquired with a bottom-mounted Gatan OneView camera.

#### Cytoskeleton compounds

34 cytoskeleton drugs including myosin II, microtubule, actin modulating compounds and one ROCK inhibitor were used to perform dose-response assay on MOLM14 WT and *SRSF2* Mut cells at concentration of 0.037μM, 0.129μM, 0.369μM, 0.997μM, 3.49μM, 9.97μM and 29.900μM in duplicates. Cell viabilities were detected as described above. The highest three concentrations were compared between WT and Mutated clones by two-way ANOVA, followed by Dunnett’s post-hoc test.

#### Splicing analysis

We used rMATS v4.0.2^42^ to assess the differential splicing landscape embedded in RNA-seq data. All the aberrant splicing events used the cut off FDR<0.05 and ΔPSI >0.1 (10%). In Figure S12D aberrant exon spliced genes (FDR<0.05) of MOLM14 and AML2 Mut cells after 2 hours exposure to RKI-1447 (0.5μM) compare with before exposure to RKI-1447 were used to perform gene enrichment analysis with clusterProfiler (v3.14.3) in R.

### Quantification and statistical analysis

In all mice engraftment figures comparison between medians was performed using the Mann−Whitney U test with FDR correction for multiple hypothesis testing. For Figures 1E and 1G, the highest three concentrations were compared between WT and Mutated clones by two-way ANOVA, followed by Dunnett’s post-hoc test (Data S6 and S10).

The p-value of the overlap of aberrant exon usage gene of all our *SRSF2* Mut cell lines and *SRSF2* mut BeatAML database were analyzed with the following code in R:

hygeo5<-function(N,m,a,b,c,h,w){

 #N=11027# total number of genes for selection

 #m=14 # number of overlaps in all three sets

 #a=1332# number of aberrant spliced genes in OCI-AML2 SRSF2 Mut

 #b=1295# number of aberrant spliced genes in MARIMO SRSF2 Mut

 #c=1025# number of aberrant spliced genes in MOLM14 SRSF2 Mut

 #h=1723# number of aberrant spliced genes in K562 SRSF2 Mut

 #w=488# number of aberrant spliced genes in BeatAML SRSF2 Mut

 d<-data.frame(0:min(a,b,c,h,w),rep(0,min(a,b,c,h,w)+1))

 for (i in 1:10000){

 A=sample(1:N,size=a,replace=FALSE)

 B=sample(1:N,size=b,replace=FALSE)

 C=sample(1:N,size=c,replace=FALSE)

 H=sample(1:N,size=h,replace=FALSE)

 W=sample(1:N,size=w,replace=FALSE)

 e<-table((C %in% A)&(C %in% B)&(C %in% H)&(C %in% W))["TRUE"]

 if(!complete.cases(e)){

 e<-0

 }

 d[e+1,2]<-d[e+1,2]+1

 }

 colnames(d)<-c("Intersect","p-value")

 d[,2]<-d[,2]/10000

 p<-sum(d[(m+1):(min(a,b,c,h,w)+1),2])

 print(d)

 return(p)

}

hygeo5(11027,14,1332,1295,1025,1723,488)

## Supplemental information

Document S1. Figures S1–S17

Data S1. Targeted amplicon sequencing of all *SRSF2* Mut cell lines and predicted off-target of sgRNA, related to Figure 1A

Data S2. Aberrant splicing events of *SRSF2* Mut cells lines, related to Figures 1A and 1B

Data S3. List of exons skipped/included (SE) genes of *SRSF2* Mut cell lines and Beat AML database, related to Figures 1A and 1B

Data S4. HTDS of 3988 chemical compounds in a single dose (10 μM), related to Figure 1D

Data S5. Dose response analysis and IC50 of 44 compounds, related to Figures 1D and 1E

Data S6. Dose response analysis, IC50, and statistics of four ROCK inhibitors on MOLM14, AML2, and MARIMO cell lines, related to Figures 1E and S1C

Data S7. Proteomics and phosphoproteomics data of MOLM14 and OCI-AML2 cells before and after exposure to RKI-1447, related to Figures 3A–3C, S2A, and S2B

Data S8. Clinical and molecular data of primary samples, related to Figure 2A

Data S9. *In vitro* liver toxicity of RKI-1447, related to Figures S3H

Data S10. GSEA of proteomic data, quantification of surface, sphericity and volume of nucleus, and statistics of cytoskeleton compounds screening, related to Figures 3B, 4B, and 4G
