# Supplementary material for: Targeting SRSF2 mutations in leukemia with RKI-1447: A strategy to impair cellular division and nuclear structure
Source: iScience. 2024 Mar 6;27(4):109443. doi: 10.1016/j.isci.2024.109443 (PMC10981050; doi:10.1016/j.isci.2024.109443)
Supplement: Document S1. Figures S1–S17 [file mmc1.pdf]

iScience, Volume *27*

Supplemental information

Targeting *SRSF2* mutations in leukemia with RKI-1447: A strategy to impair cellular division and nuclear structure

Minhua Su, Tom Fleischer, Inna Grosheva, Melanie Bokstad Horev, Malgorzata Olszewska, Camilla Ciolli Mattioli, Haim Barr, Alexander Plotnikov, Silvia Carvalho, Yoni Moskovich, Mark D. Minden, Noa Chapal-Ilani, Alexander Wainstein, Eirini P. Papapetrou, Nili Dezorella, Tao Cheng, Nathali Kaushansky, Benjamin Geiger, and Liran I. Shlush

Fig. S1

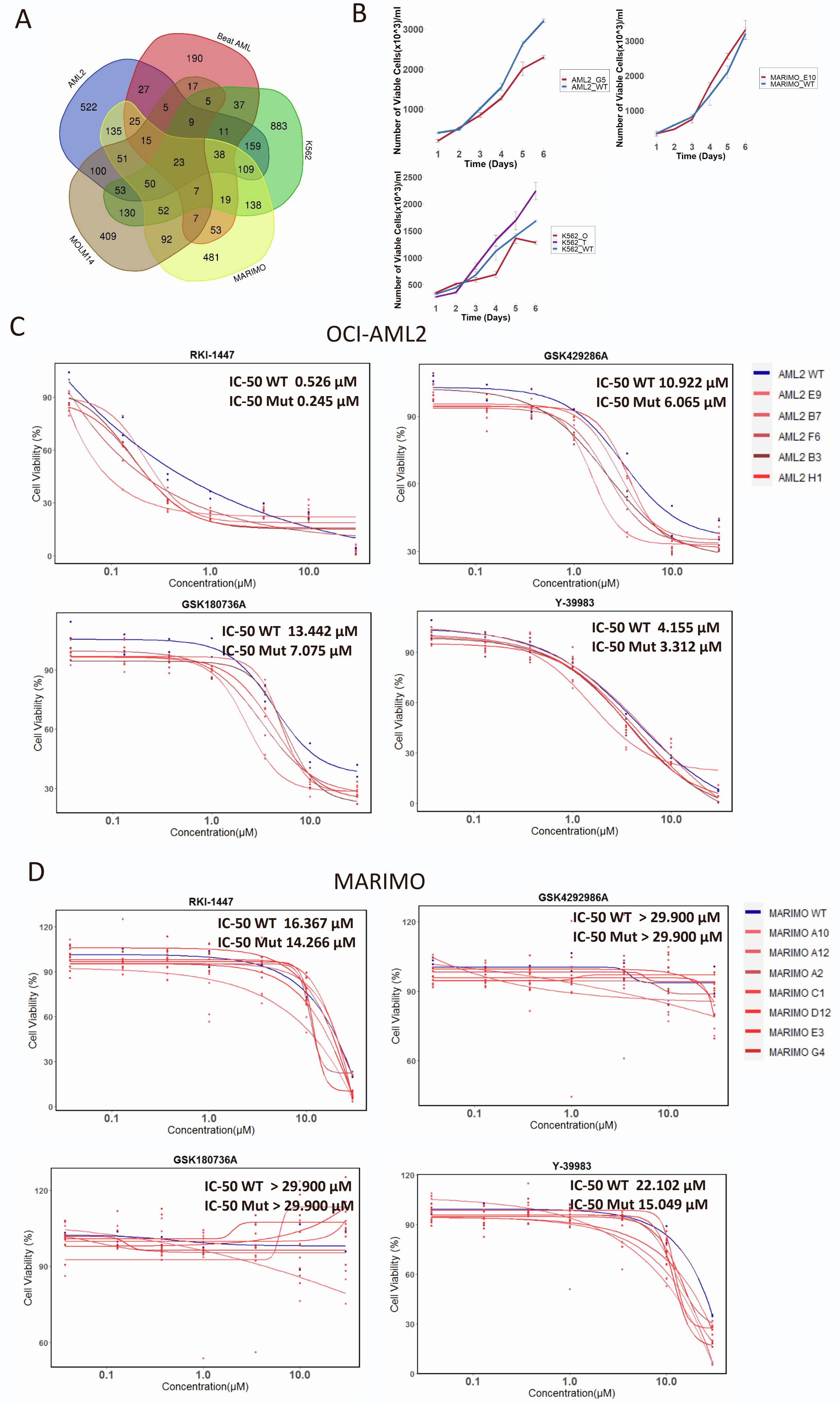
A
AML2
Beat AML
K562
MARIMO
MOLM14
B
Number of Viable Cells(x10^3)/ml
Time (Days)
AML2_G5
AML2_WT
MARIMO_E10
MARIMO_WT
K562_O
K562_T
K562_WT
C
OCI-AML2
RKI-1447
IC-50 WT 0.526 µM
IC-50 Mut 0.245 µM
GSK429286A
IC-50 WT 10.922 µM
IC-50 Mut 6.065 µM
GSK180736A
IC-50 WT 13.442 µM
IC-50 Mut 7.075 µM
Y-39983
IC-50 WT 4.155 µM
IC-50 Mut 3.312 µM
Cell Viability (%)
Concentration(µM)
AML2 WT
AML2 E9
AML2 B7
AML2 F6
AML2 B3
AML2 H1
D
MARIMO
RKI-1447
IC-50 WT 16.367 µM
IC-50 Mut 14.266 µM
GSK4292986A
IC-50 WT > 29.900 µM
IC-50 Mut > 29.900 µM
GSK180736A
IC-50 WT > 29.900 µM
IC-50 Mut > 29.900 µM
Y-39983
IC-50 WT 22.102 µM
IC-50 Mut 15.049 µM
MARIMO WT
MARIMO A10
MARIMO A12
MARIMO A2
MARIMO C1
MARIMO D12
MARIMO E3
MARIMO G4

Fig. S1: **Aberrant exons spliced genes and cell proliferation growth and dose-response curve of ROCKi**
(**A**) Venn diagram of aberrant exon usage (SE/IE) identified significant overlap (p<10e6, hypergeometric test, see **supplementary methods)** of 23 genes with aberrant exon splicing in all *SRSF2* Mut cell lines and samples. (**B**) The cell proliferation growth curves of *SRSF2* WT/Mut OCI-AML2, MARIMIO and K562 SRSF2 WT and Mutated (G5, E10, O and T respectively) cell lines. Cells were seeded in T25 flask at the concentration of 300,000 cells/ml and the cells were counted manually each day for six days. Technical duplicates of each line and each day. (**C**) Dose-respond curve of *SRSF2* WT and *SRSF2* Mut OCI-AML2 cells (AML2 E9, AML2 B7, AML2 F6, AML2 B3, AML2 H1) on RKI-1447, GSK429286A, GSK180736A and Y-39983 at concentration of 0.037μM, 0.129μM, 0.369μM, 0.997μM, 29.900μM. **(D)** Dose-respond curve of *SRSF2* WT/Mut MARIMO cells (MARIMO A10, MARIMO A12, MARIMO A2, MARIMO C1, MARIMO D12, MARIMO E3, MARIMO G4) on RKI-1447, GSK429286A, GSK180736A and Y-39983 at concentration of 0.037μM, 0.129μM, 0.369μM, 0.997μM, 29.900μM. Mean of IC-50 value of all mutant clones and WT were shown for each compound. The highest three concentrations were compared between WT and mutant clones by two-way ANOVA, followed by Dunnett's post-hoc test (**Data files S6**).

Fig. S2

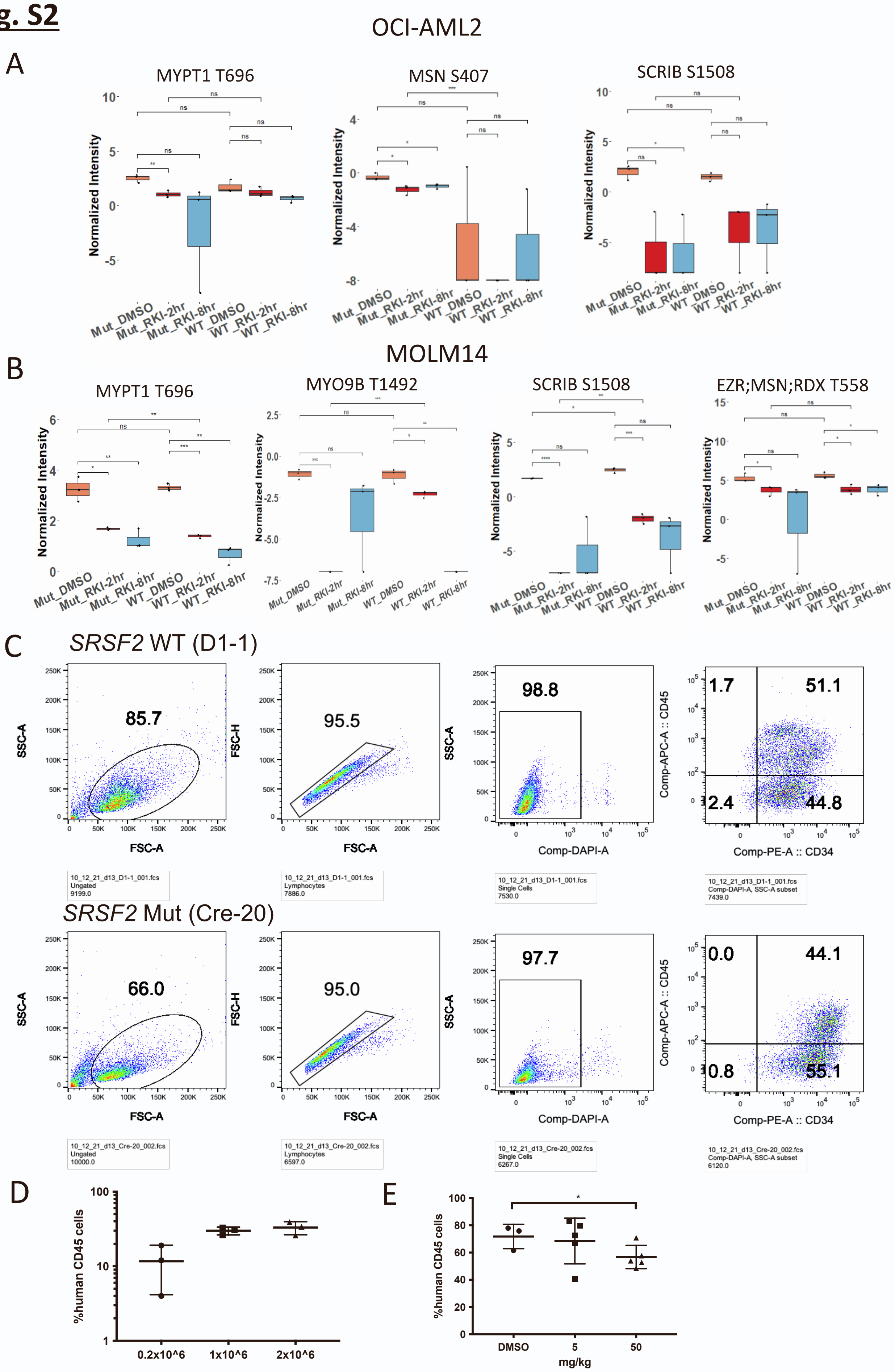

Fig. S2: Phosphoproteomics of *SRSF2* WT/Mut cells and flow profiles of isogenic *SRSF2* WT and P95L iPSC-derived HSPCs and engraftment of MOLM14 *SRSF2* Mut cells in NSG mice. Phosphoproteomics was performed on *SRSF2* WT/Mut MOLM14 and AML2 cells. Cells were treated with DMSO or RKI-1447 (0.5μM) for two hours and eight hours. Normalized intensity of phosphorylation level of several downstream targets of ROCKs, MYPT1 T696, MSN S407, SCRIB S1508, MYO9B T1492, SCRIB S1508 and EZR;MSN;RDX T558 were shown in AML2 cells (**A**) and MOLM14 cells (**B**). (**C**)Flow profiles Isogenic *SRSF2* WT (D1-1) and P95L (Cre20) iPSC-derived HSPCs on day12 differentiation. CD34 and CD45 expression at days 12 were shown in far right column. Cells were then cultured with DMSO or RKI-1447 (3μM) for 48 hours and viability was measured with CellTiter-Glo (**Fig. 1G**). **(B)** NSG mice were injected with 0.2*10^6, 1*10^6, and 2*10^6 MOLM14 *SRSF2* Mut cells by intravenous (IV) injection (N=3 for each group) to calibrate the number of cells for MOLM14 *SRSF2* xenograft model. Mice were sacrificed after 8-week transplantation to determine the amount of MOLM14 *SRSF2* Mut cells for our *in vivo* model. (**C**) NSG mice were treated with vehicle (DMSO) or RKI-1447 (5mg/kg or 50mg/kg) by i.p. for 21 days after 5-week transplantation of one million *SRSF2* Mut MOLM14 cells (IV) to calibrate the efficacious dosage of RKI-1447 *in vivo*. Mice were sacrificed and BM cells was flashed from tibia/femur. The percentage of human CD45+ (hCD45) cells in engrafted murine bone marrow is shown after staining for hCD45 and analyzed by flow cytometry. Mann−Whitney U test with FDR correction for multiple hypothesis testing, *P<0.05; **P<0.005; ***P<0.0005.

Fig. S3

A

Mouse No.	VAF	AAChange.refGene
209945_DMSO_16	0.481	c.C284G:p.P95R
209945_DMSO_17	0.284	c.C284G:p.P95R
209945_DMSO_19	0.293	c.C284G:p.P95R
209945_DMSO_20	0.488	c.C284G:p.P95R
209945_RKI_23	0.419	c.C284G:p.P95R
209945_RKI_27	0.46	c.C284G:p.P95R
209945_RKI_28	0.49	c.C284G:p.P95R
830163_DMSO_6	0.0706	c.C284G:p.P95R
830163_RKI_12	0.0284	c.C284G:p.P95R
800667_DMSO_10	0.551	c.C284A:p.P95H
800667_DMSO_11	0.5371	c.C284A:p.P95H
800667_DMSO_7	0.5426	c.C284A:p.P95H
800667_DMSO_8	0.5267	c.C284A:p.P95H
800667_DMSO_9	0.5442	c.C284A:p.P95H
800667_RKI_12	0.5467	c.C284A:p.P95H
800667_RKI_13	0.545	c.C284A:p.P95H
800667_RKI_14	0.5363	c.C284A:p.P95H
800667_RKI_15	0.5435	c.C284A:p.P95H
800667_RKI_16	0.5345	c.C284A:p.P95H

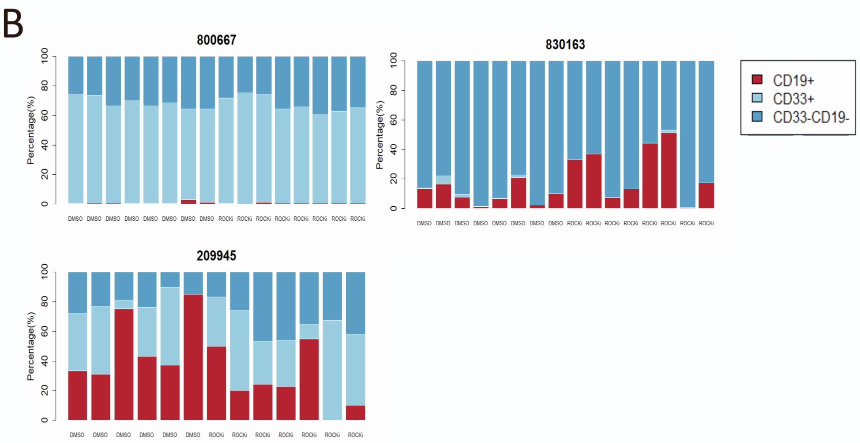

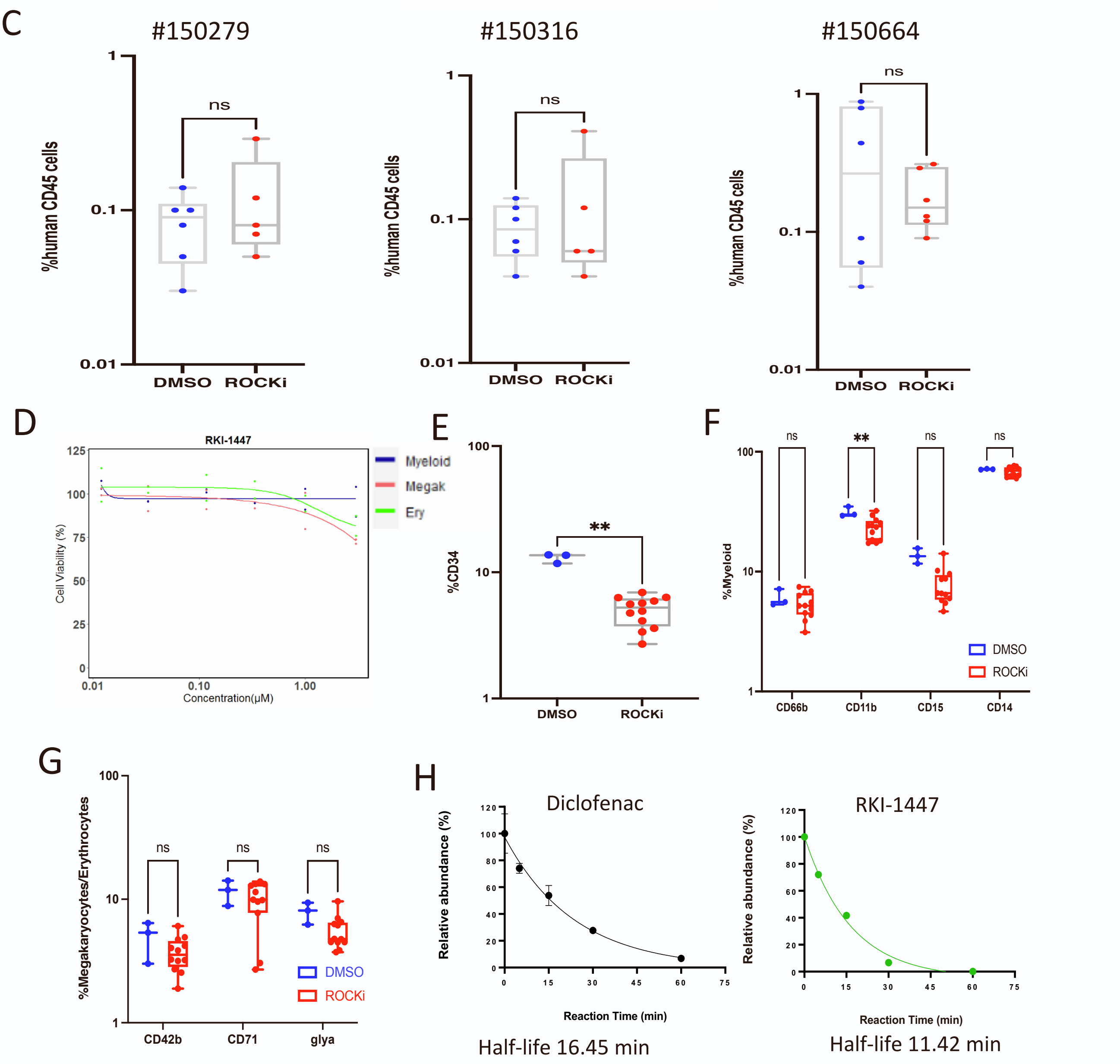

Fig. S3: VAF of *SRSF2* Mutations of CD45+ grafts, types of engraftment and the effect of RKI-1447 on CD34+ cells *in vivo*. (**A**) CD45+ cells from the grafts (**Fig. 2A**) were isolated and the DNA of *SRSF2* P95 region were sequenced by targeted amplicon sequencing. VAF (variant allele frequency) is indicated with bar plot in each mouse. (**B**) The percentage of CD19+, CD33+ and CD19-CD33- human CD45+ (hCD45) cells in engrafted murine bone marrow were measured with by flow cytometry to determine myeloid or multi lineage engraftment of *SRSF2* Mut (#800667, #830163 and #209945) AML samples. (**C**) CD3 depleted frozen PBMC from three *SRSF2* WT AML samples were injected into NSG mice (#150279, #150316 and #150664) intrafemorally (i.f.). On day 35 the animals were randomized to RKI-1447 or a carrier control. RKI-1447 is administered i.p. at a dose of 50mg/kg daily for 21 days. On day 56 mice were sacrificed and analyzed for human CD45+ (hCD45) cells engraftment by flow cytometry. Mann−Whitney U test with FDR correction for multiple hypothesis testing, *P<0.05; **P<0.005; ***P<0.0005. (**D**) 1000-2000 healthy CD34+ cells were cultured with myeloid, erythroid and megakaryocytic lineage differentiation cytokines for 7 days, followed by addition of RKI-1447 (3µM, 1µM, 0.33µM, 0.11µM, 0.04µM, 0.01µM) for 48 hours. Cell viability was detected with CellTiter-Glo assay and normalized to DMSO control. **(E)** Percentage of CD34+ cells was detected by flow under myeloid differentiation condition. (**F**) Myeloid lineage cells were detected with CD66b, CD11b, CD15, CD14; (**G**) Megakaryocytes were detected with CD42b; Erythrocytes were detected with CD71 and GlyA. Mann−Whitney U test with FDR correction for multiple hypothesis testing, *P<0.05; **P<0.005; ***P<0.0005. **(H)** RKI-1447 (1 µM) was incubated with human liver microsomes (HLM) and NADPH at 37 degrees for a time course using thin-walled PCR tubes. Compounds are quantified by LC/MS. Data was normalized to percent of input compound in buffer without microsomes and time-course was plotted with Graphpad Prism.

Fig. S4

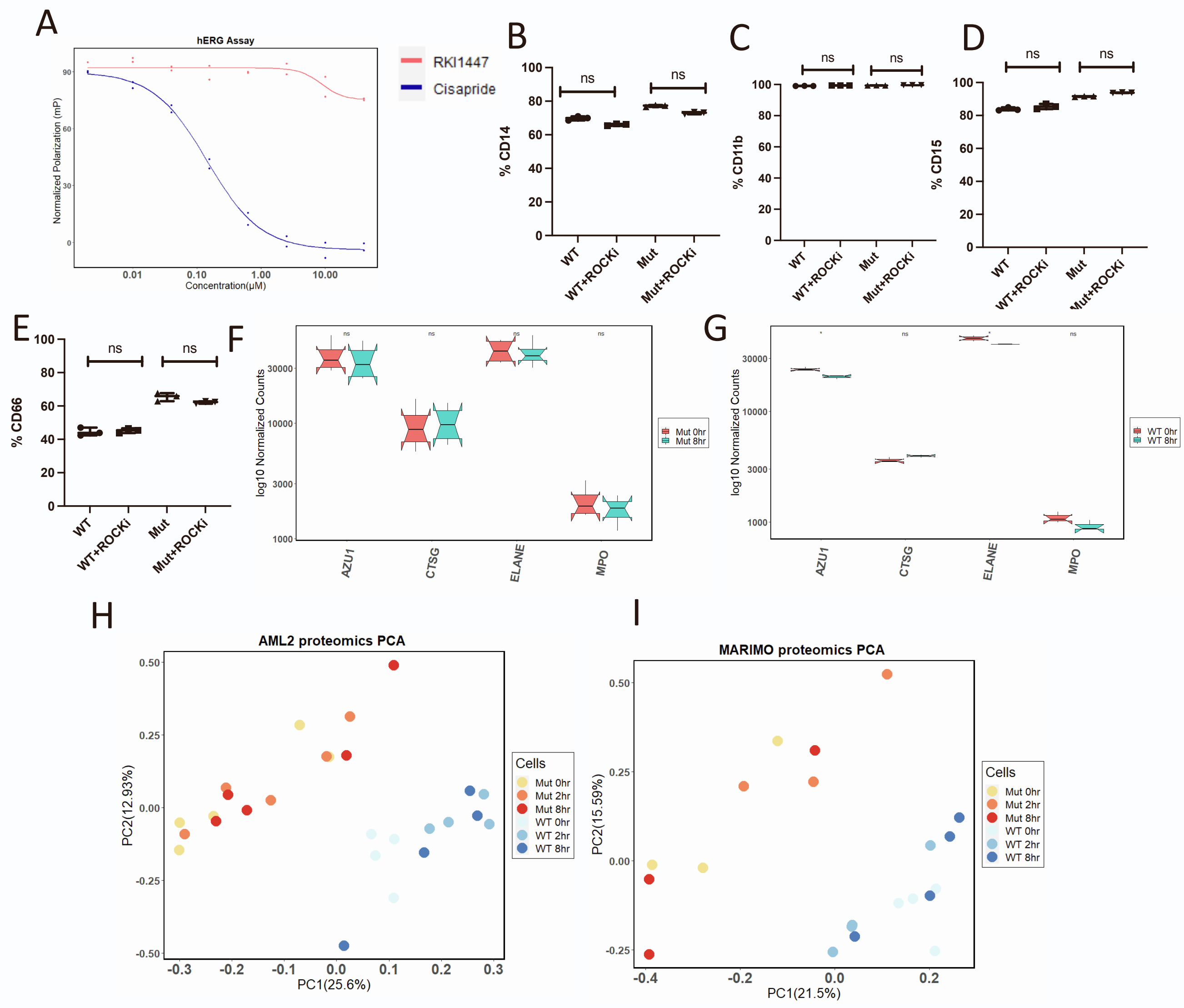

Fig. S4: hERG assay of RKI-1447, differentiation of MOLM14 cells under exposure to RKI-1447, and principal component analysis (PCA) of protein expression of AML2 and MARIMO cells. (A) The cardiotoxic effect was measured by the blockage of the human ether-a-go-go-related gene (hERG) potassium channel by Predictor™ hERG Fluorescence Polarization Assay. The assay is performed with different concentration (0.01μM, 0.04μM, 0.33μM, 1μM and 3μM) of RKI-1447 and Cisapride (positive control). Y axis is polarization (mP) normalized (where full binding is 0, and no binding is 100%). **(B-E)**, Differentiation of *SRSF2* WT and Mut MOLM14 cells are detected with different surface markers (CD14, CD11b, CD15, CD66) by flow cytometry after 24hr addition of RKI-1447 (0.5μM). **(F)** Gene expression of neutrophil differentiation markers (*AZU1*, *CTSG*, *ELANE*, *MPO*) of *SRSF2* Mut MOLM14 cells before and after 8-hour exposure to RKI-1447 (0. 5μM); **(G)** gene expression of neutrophil differentiation markers (*AZU1*, *CTSG*, *ELANE*, *MPO*) of *SRSF2* WT MOLM14 cells before and after 8-hour exposure to RKI-1447 (0.5μM). **(H)** Principal component analysis plot of protein expression of *SRSF2* WT/Mut AML2 cells before (0hr) and after 2hr and 8hr exposure to RKI-1447 (0.5μM). **(I)** Principal component analysis plot of protein expression of MARIMO *SRSF2* WT/Mut cells before (0hr) and after 2hr and 8hr exposure to RKI-1447 (0.5μM).

Fig. S5

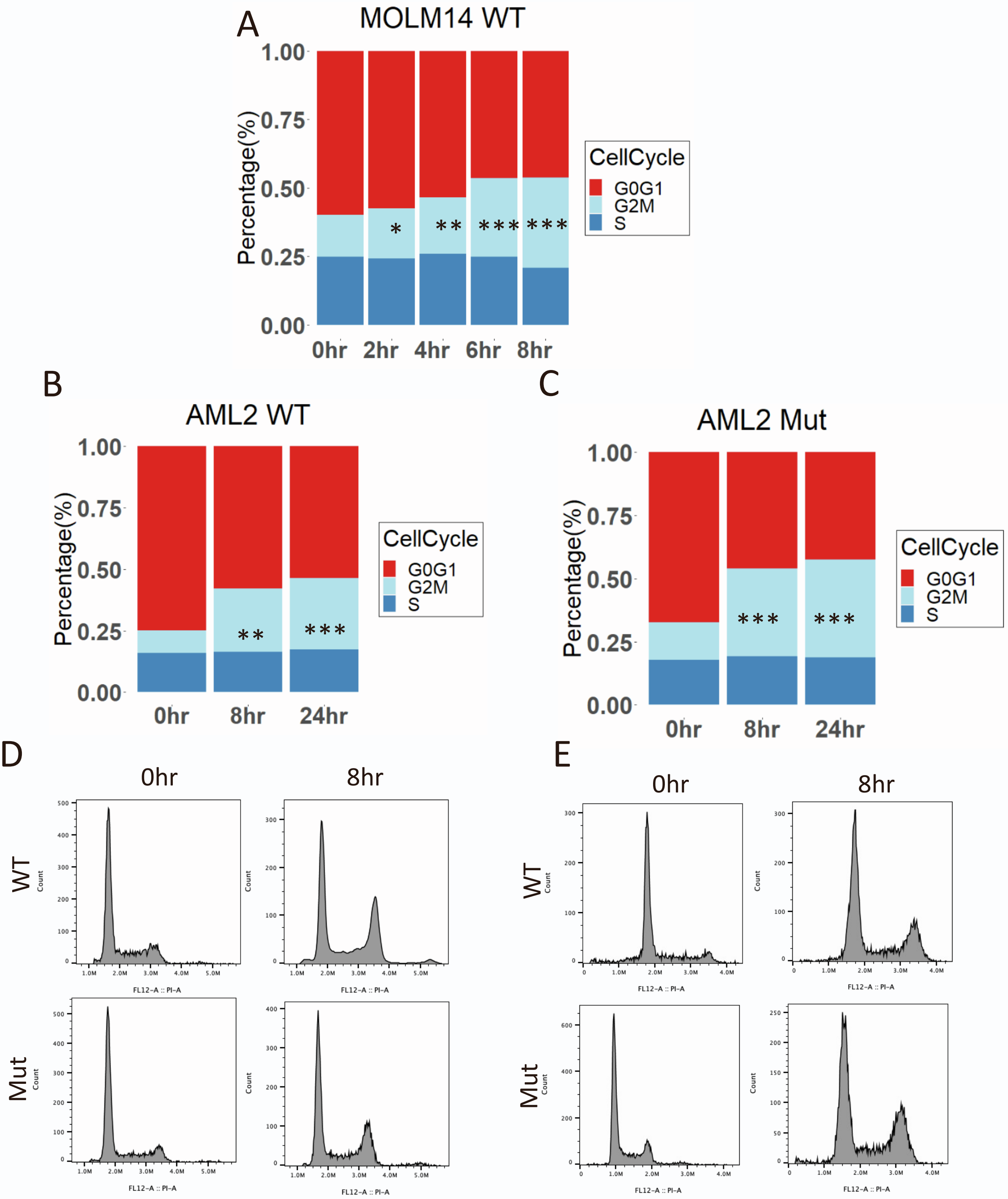

Fig.S5: **Cell cycle analysis of MOLM14 WT and AML2 WT/Mut cells before and after exposure to RKI-1447. (A)** Cell cycle of MOLM14 WT before and after 2hr, 4hr, 6hr, and 8hr exposure to RKI-1447(0.5μM). **(B-C)**. Cell cycle of AML2 WT (**B**) and OCI-AML2 Mut (**C**) before and after 8hr, and 24hr exposure to RKI-1447 (0.5μM). Different cell cycles are indicated with different colors in the plot. Statistical analysis was performed using two-sided t-tests; *P<0.05; **P<0.005; ***P<0.0005. (**D-E**) Representative histogram of linear PI content in *SRSF2*-mutant and WT MOLM14 (**D**) and OCI-AML2 (**E**) cells incubated with either DMSO or RKI-1447.

Fig. S6

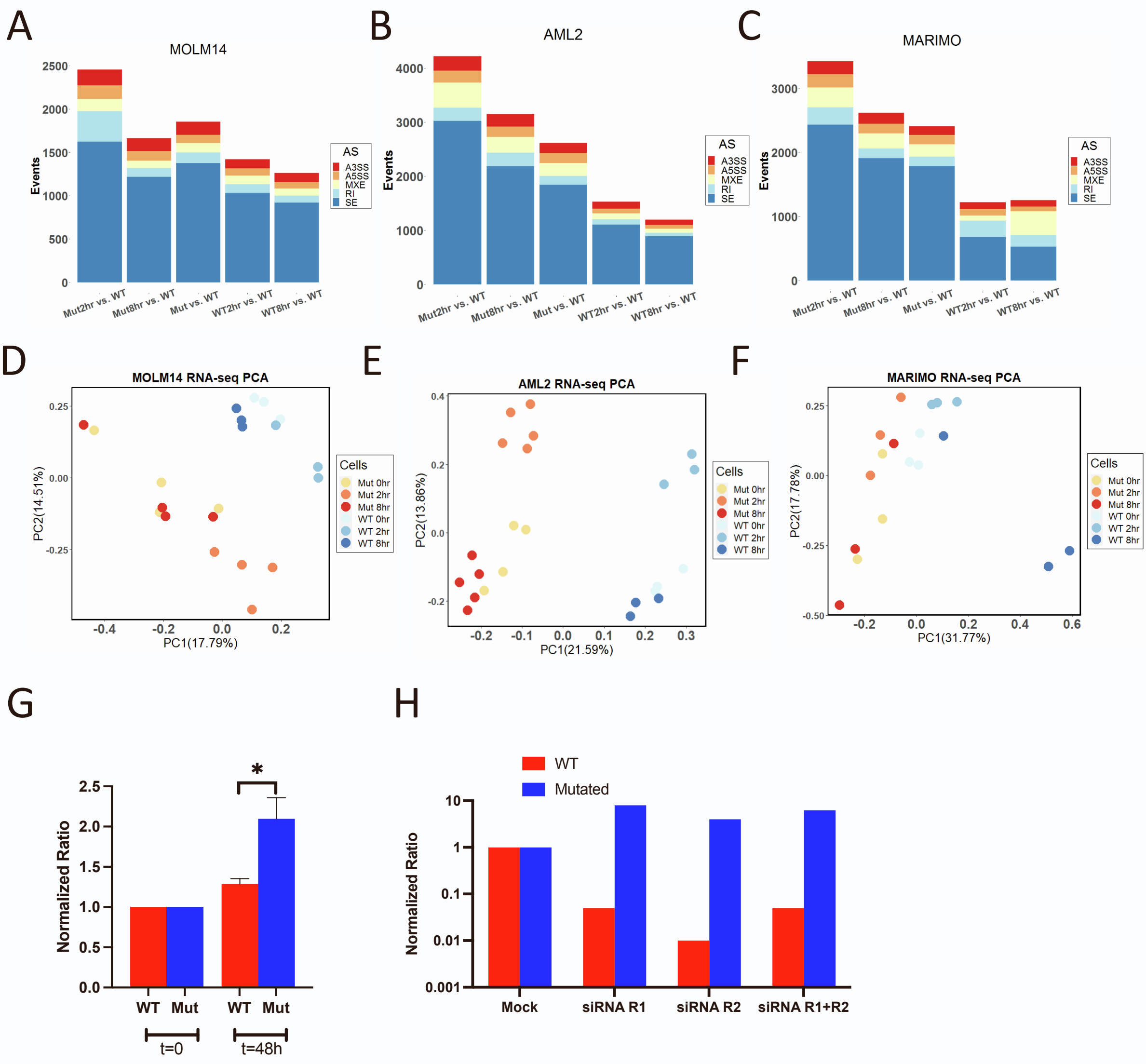

Fig.S6: Number of aberrant exon splicing events after exposure to RKI-1447 and principal component analysis of mRNA expression of MOLM14, AML2 and MARIMO cells before and after exposure to RKI-1447. RNA-seq was performed for *SRSF2* Mut and WT MOLM14, AML2, MARIMO before and after two and eight hours exposure to RKI-1447 (0.5µM). Splicing event were filter by FDR<0.05, Δ|PSI|> 10%. **(A)** Numbers of aberrant splicing events of MOLM14 *SRSF2* Mut with addition of RKI-1447 2hr or 8hr vs. MOLM14 WT, MOLM14 *SRSF2* Mut vs. MOLM14 WT, MOLM14 WT with addition of RKI-1447 2hr or 8hr vs. MOLM14 WT. **(B)** Numbers of aberrant splicing events analysis of OCI-AML2 *SRSF2* Mut with addition of RKI-1447 2hr or 8hr vs. OCI-AML2 WT, OCI-AML2 *SRSF2* Mut vs. OCI-AML2 WT, OCI-AML2 WT with addition of RKI-1447 2hr or 8hr vs. OCI-AML2 WT. **(C)** Numbers of aberrant splicing events of MARIMO *SRSF2* Mut with addition of RKI-1447 2hr and 8hr vs. MARIMO WT, MARIMO *SRSF2* Mut vs. MARIMO WT, MARIMO WT with addition of RKI-1447 2hr or 8hr vs. MARIMO WT. Alternative 3’ splice sites (A3SS), alternative 5’ splice sites (A5SS), mutually exclusive exons (MXE), retained intron (RI) and skipped/included exons (SE), aberrant splicing (AS). **(D-F)** Principal component analysis plot of mRNA expression of MOLM14 (**D**), AML2 (**E**) and MARIMO (**F**) *SRSF2* WT/Mut cells before (0hr), after 2hr or 8hr exposure to RKI-1447 (0.5µM). (**G**) The percentages of late apoptosis (Annexin V+/PI+) in MOLM14 *SRSF2* WT/Mut cells, after 48 hours of exposure to RKI-1447 (0.5µM), were normalized to the percentages at 0 hours. Data averages were calculated from three replicates, and the results are expressed as means ± standard error (s.e.), N=3. Statistical analysis was performed using two-sided t-tests, *P<0.05. (**H**) The percentages of late apoptosis (Annexin V+/PI+) in SRSF2 WT/Mut MOLM14 cells, after 48 hours of siRNA knockdown of rock1 and/or rock2 (250nM), were normalized to the percentages at 0 hours.

Fig. S7

A

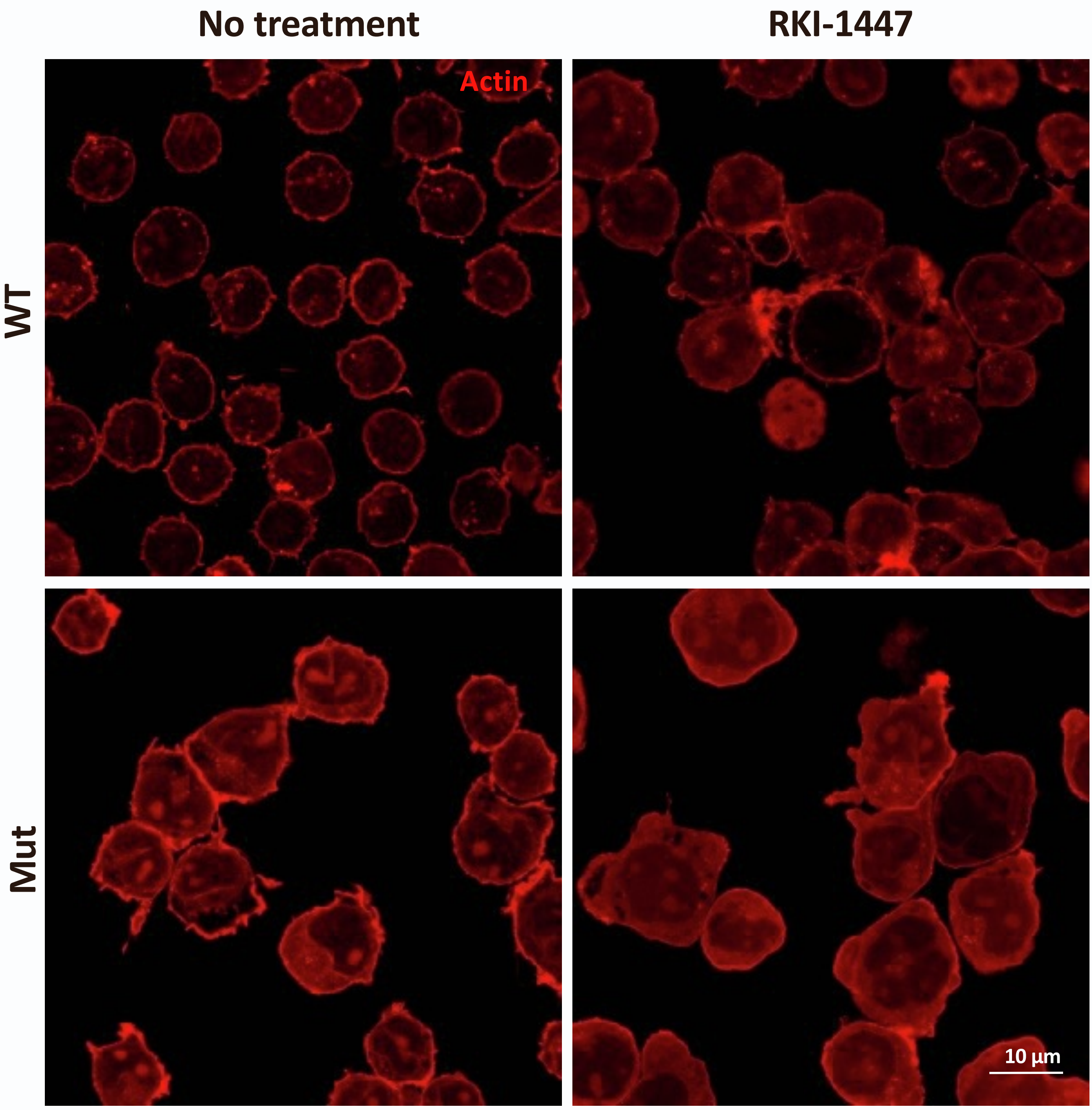

B

Sample	CY actin (MI)	NU actin (MI)	Ratio NU actin/CY actin
WT NT	1809	933	0.51
Mut NT	3230	2788	0.86
WT RKI-1447	1785	1208	0.68
Mut RKI-1447	5773	3844	0.67

Fig. S7: F-actin staining of *SRSF2* WT and Mut MOLM14 cells before and after exposure to RKI-1447. *SRSF2* WT/Mut MOLM14 cells, as indicated, were either left untreated or treated with RKI-1447 (0.5μM) for 24 hours before fixation. **(A)** Cells were labeled with fluorescent phalloidin to visualize actin (shown in red). 3D volumes were taken on the Leica SP8 scanning confocal microscope, represented slices corresponding to mid-plane of cells are shown. (**B**), Quantification of the nuclear vs cytoplasmic actin was performed, with N values as follows N = 36 for WT, N= 26 for mut, N=29 for WT + RKI-1447 and N=26 for Mut + RKI-1447. Note, that the nuclear-to- cytoplasmic actin ratio was higher in mutant compared to WT, and higher in RKI-1447-treated WT cells, compared to non-treated WT nuclei.

Fig. S8

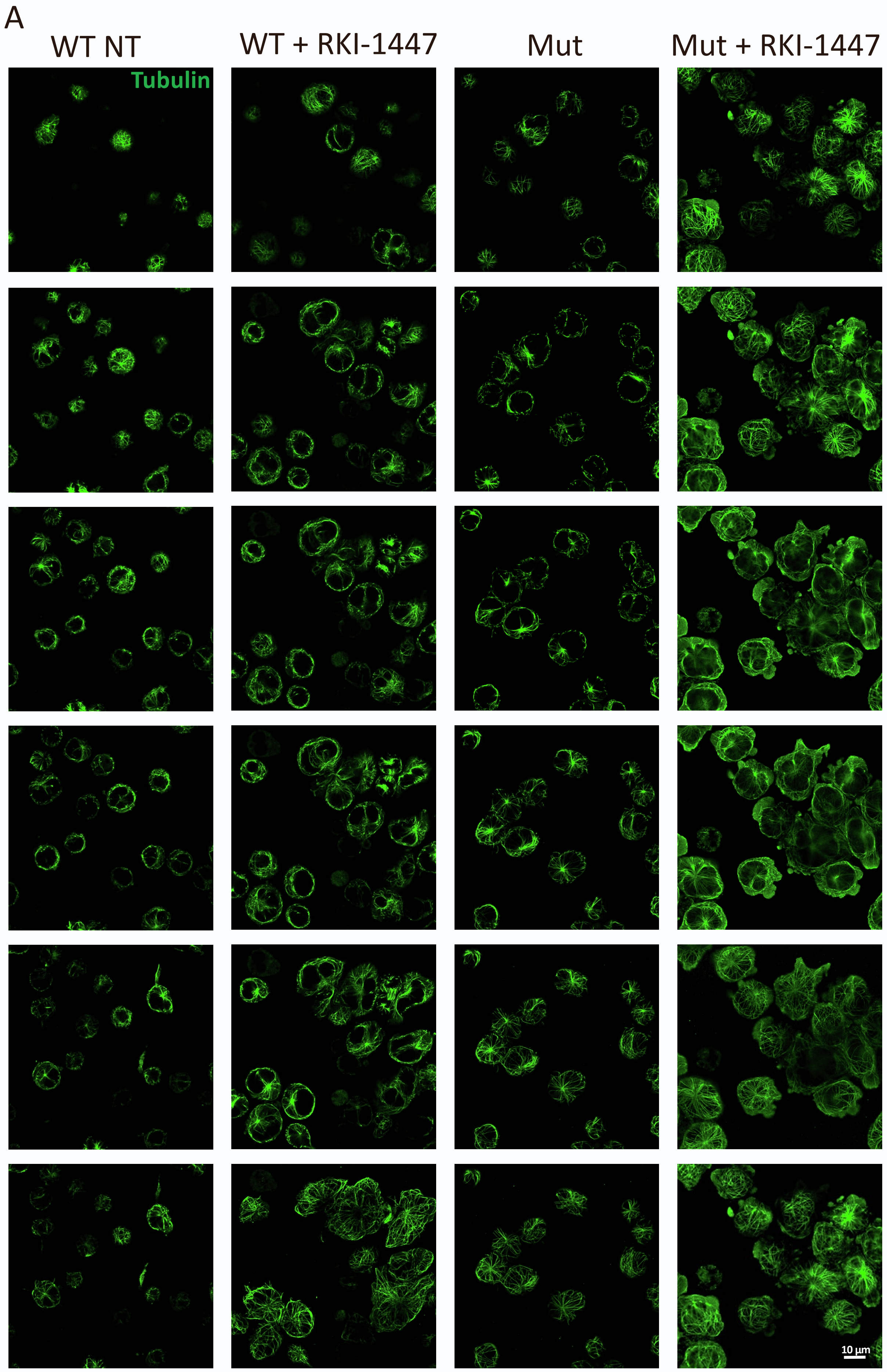

Fig. S8: Z-stack confocal images of microtubules staining of *SRSF2* WT/Mut MOLM14 cells before and after exposure to RKI-1447. (**A**) *SRSF2* WT/Mut MOLM14 cells, as indicated, were either left untreated or treated RKI-1447 (0.5 μM) for 24 hours before fixation. Cells were labeled with anti-α-tubulin antibodies to label microtubules (shown in green). The z-stack images were acquired using the Leica SP8 scanning confocal microscope (shown in green). The confocal slices are represented from top to bottom on the figure and range from top to bottom by the sample. The z volume was divided in six equal distances from the first slice chosen to the last. The slices are approximately 1.5 μm apart. Note, the prominence of microtubule bundles, especially in mutant cells upon RKI-1447 treatment. See also **Fig. 4A**.

Fig. S9

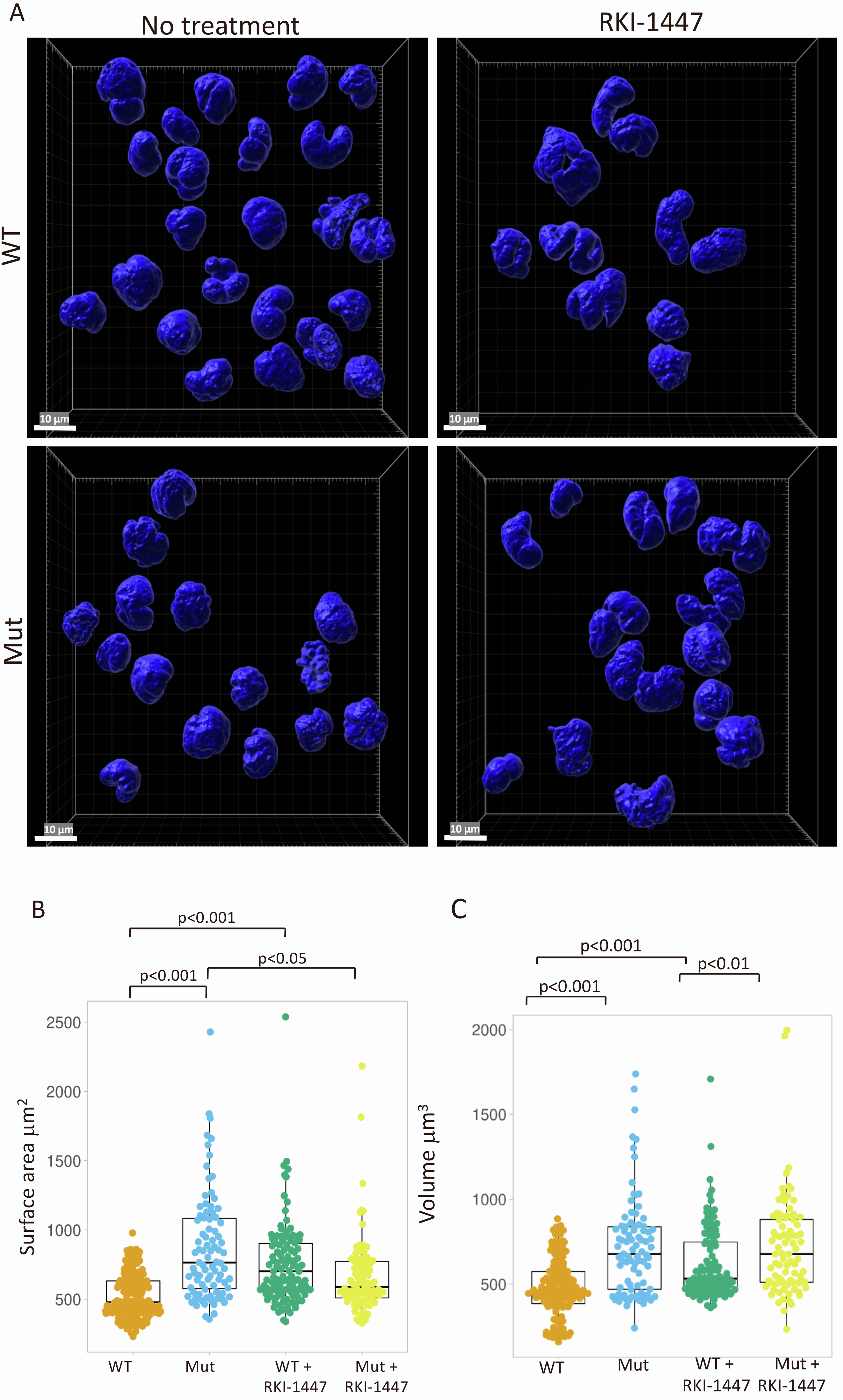
A
No treatment
RKI-1447
WT
Mut
10 µm
B
p<0.001
p<0.001
p<0.05
Surface area µm²
2500
2000
1500
1000
500
WT
Mut
WT + RKI-1447
Mut + RKI-1447
C
p<0.001
p<0.001
p<0.01
Volume µm³
2000
1500
1000
500
WT
Mut
WT + RKI-1447
Mut + RKI-1447

Fig.S9: 3D rendering of nucleus of *SRSF2* WT/Mut MOLM14 cells before and after exposure to RKI-1447. (**A**) *SRSF2* WT and Mut MOLM14 cells, as indicated, were either left untreated or treated with RKI-1447 (0.5µM) for 24 hours before fixation. Cells were labeled with DAPI to visualize nuclear morphology imaged on the Olympus confocal microscope as described in Methods. 3D volumes were subjected to 3D rendering using IMARIS software. Rendered surfaces are shown in dark blue. **(B-C)** Morphometric surface area (**B**) and rendered volume (**C**) characteristics of nucleus were extracted and plotted in the box and whiskers format. Midline corresponds to median value, box includes central 50% of distribution, and whiskers correspond to upper and bottom 25%. Note, that bearing the mutation as well as treatment with RKI-1447 causes statistically significant increase in nuclear area. N= 114 for WT, N=126 for WT+RKI-1447, N=116 for Mut, N=119 for Mut+RKI-1447. The two-sample T-test with two-tailed distribution was used for evaluation of statistical significance.

Fig. S10

A

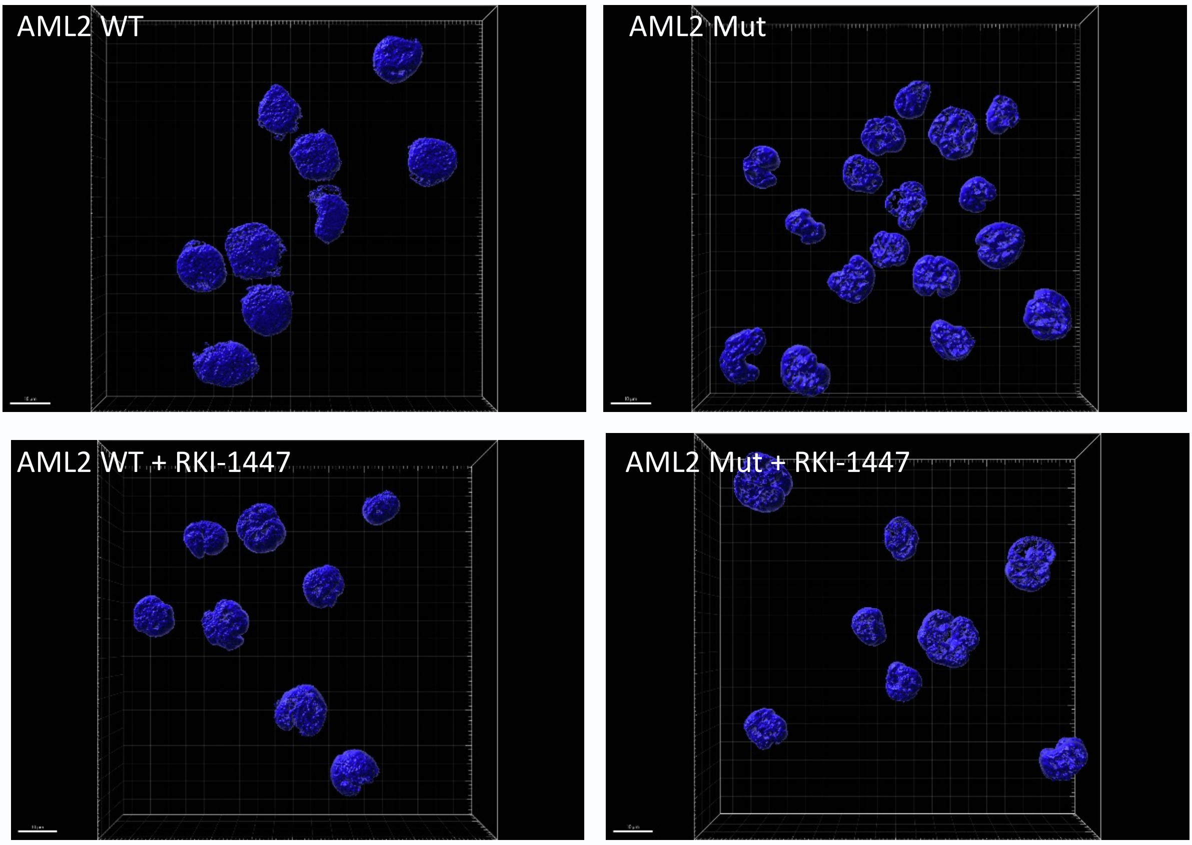
AML2 WT
AML2 Mut
AML2 WT + RKI-1447
AML2 Mut + RKI-1447

B

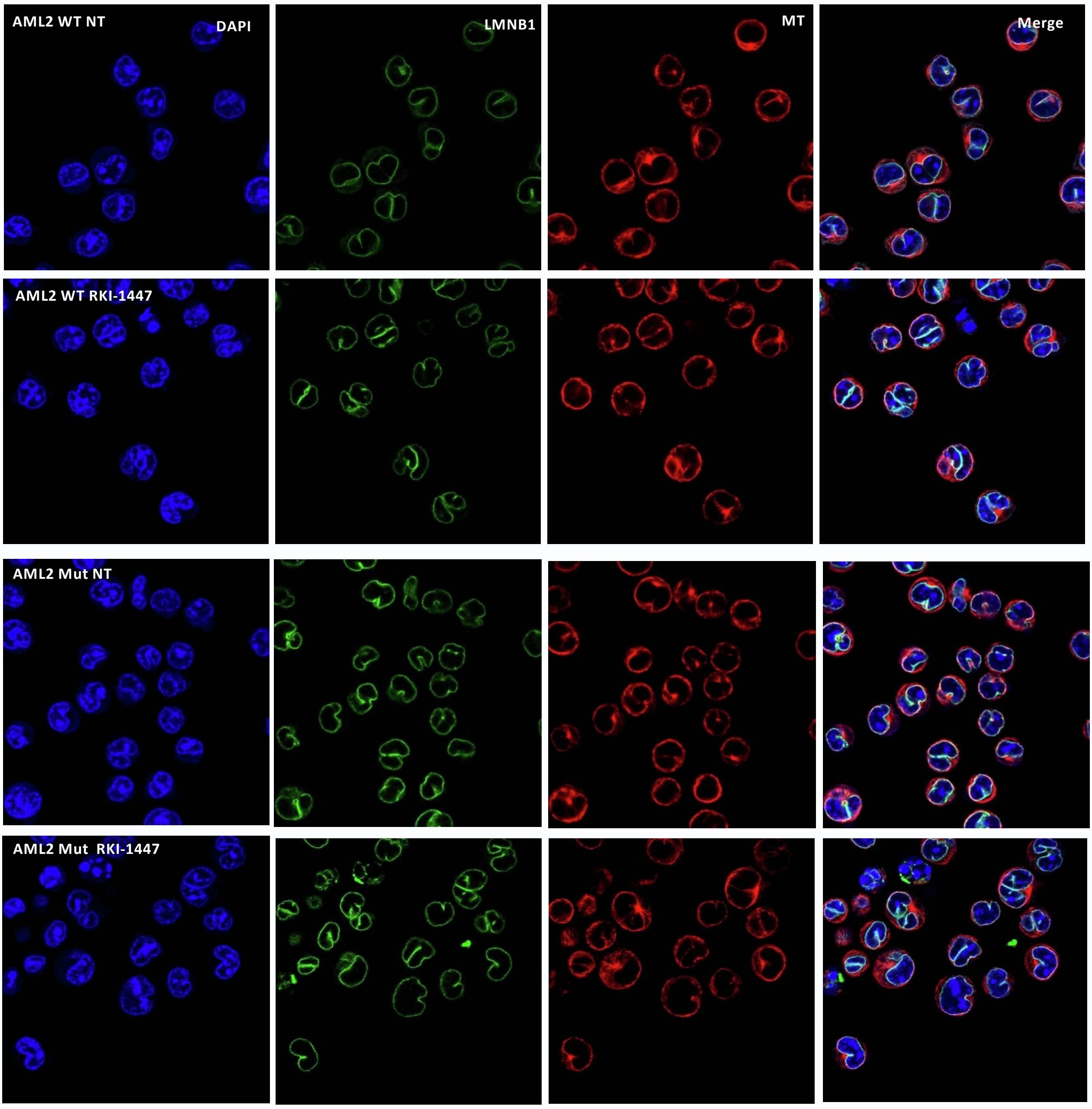
AML2 WT NT
DAPI
LMNB1
MT
Merge
AML2 WT RKI-1447
AML2 Mut NT
AML2 Mut RKI-1447

Fig. S10: 3D rendering of nucleus and confocal images of *SRSF2* WT/Mut OCI-AML2 cells before and after exposure to RKI-1447. *SRSF2* WT/Mut OCI-AML2 cells, untreated or treated with RKI-1447 (0.5µM) for 24 hours before fixation, were acquired using the Leica SP8 scanning confocal microscope. **(A)** Nuclei were labeled with DAPI to visualize nuclear morphology imaged on the Olympus confocal microscope as described in Supplementary Methods. 3D volumes were subjected to 3D rendering using IMARIS software. Rendered surfaces are shown in dark blue. Sphericity of the nucleus were measured and presented in **Fig.4B. (B)** Cells were labeled with anti-LaminB1 antibodies to outline nuclear membrane (shown in green) and anti-α-tubulin antibodies to label microtubules (shown in red), and nucleus were labeled with DAPI. Representative confocal slices corresponding to the middle plane of the cells are shown.

Fig. S11

A

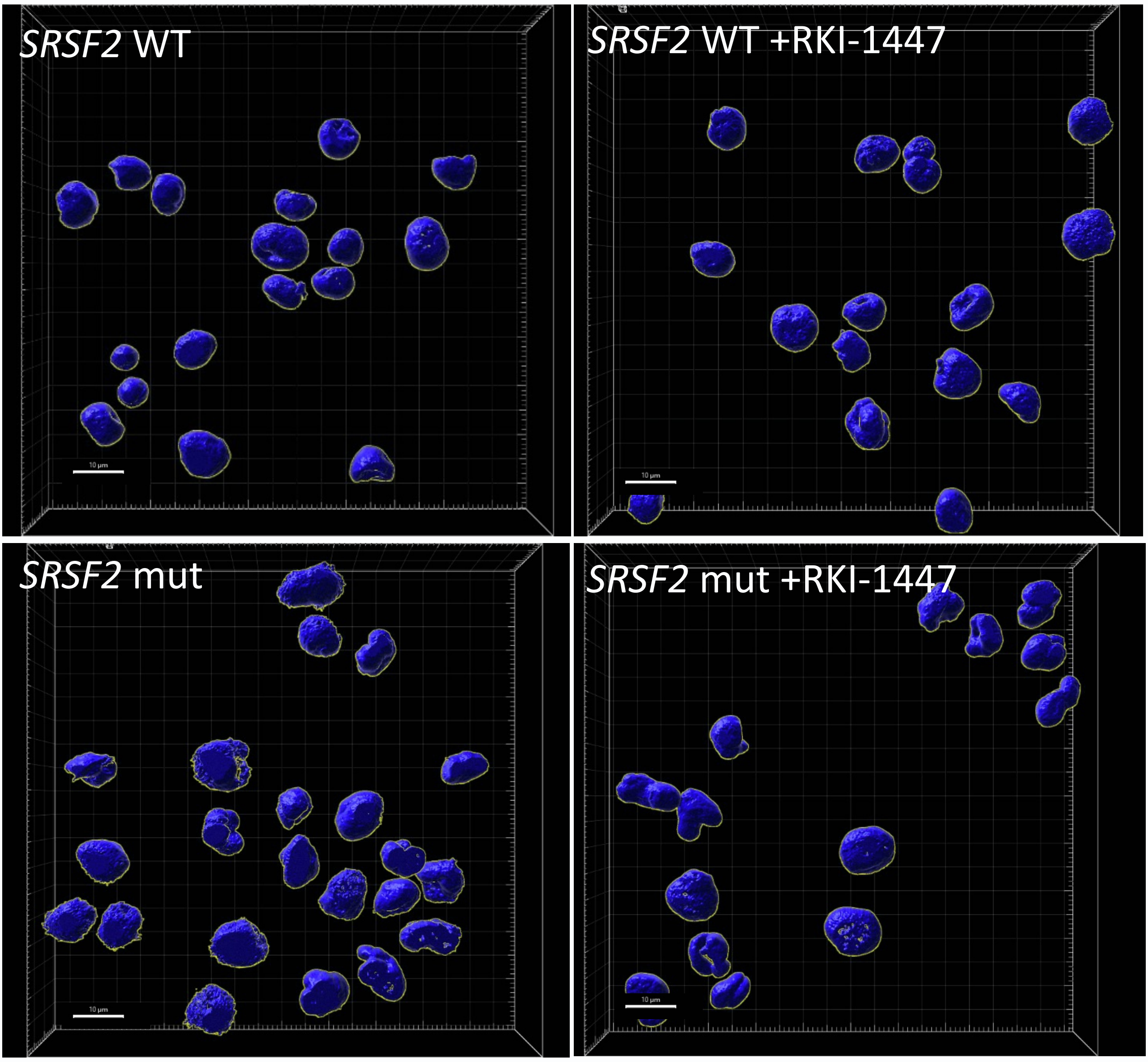

Fig. S11: 3D rendering of nucleus of *SRSF2* WT/Mut primary cells before and after exposure to RKI-1447. (A) One *SRSF2* Mut primary AML sample (#830163) and one *SRSF2* WT primary AML sample (#150279), as indicated, were either left untreated or treated with RKI-1447 (1μM) for 72 hours before fixation. Nuclei were labeled with DAPI to visualize nuclear morphology imaged on the Olympus confocal microscope as described in Methods, 3D volumes were subjected to 3D rendering using IMARIS software. Rendered surfaces are shown in dark blue. Note, that treatment with RKI-1447 causes statistically significant decrease nuclear sphericity in *SRSF2* Mut primary AML sample, see **Fig. 4B**. The two-sample T-test with two-tailed distribution was used for evaluation of statistical significance.

Fig. S12

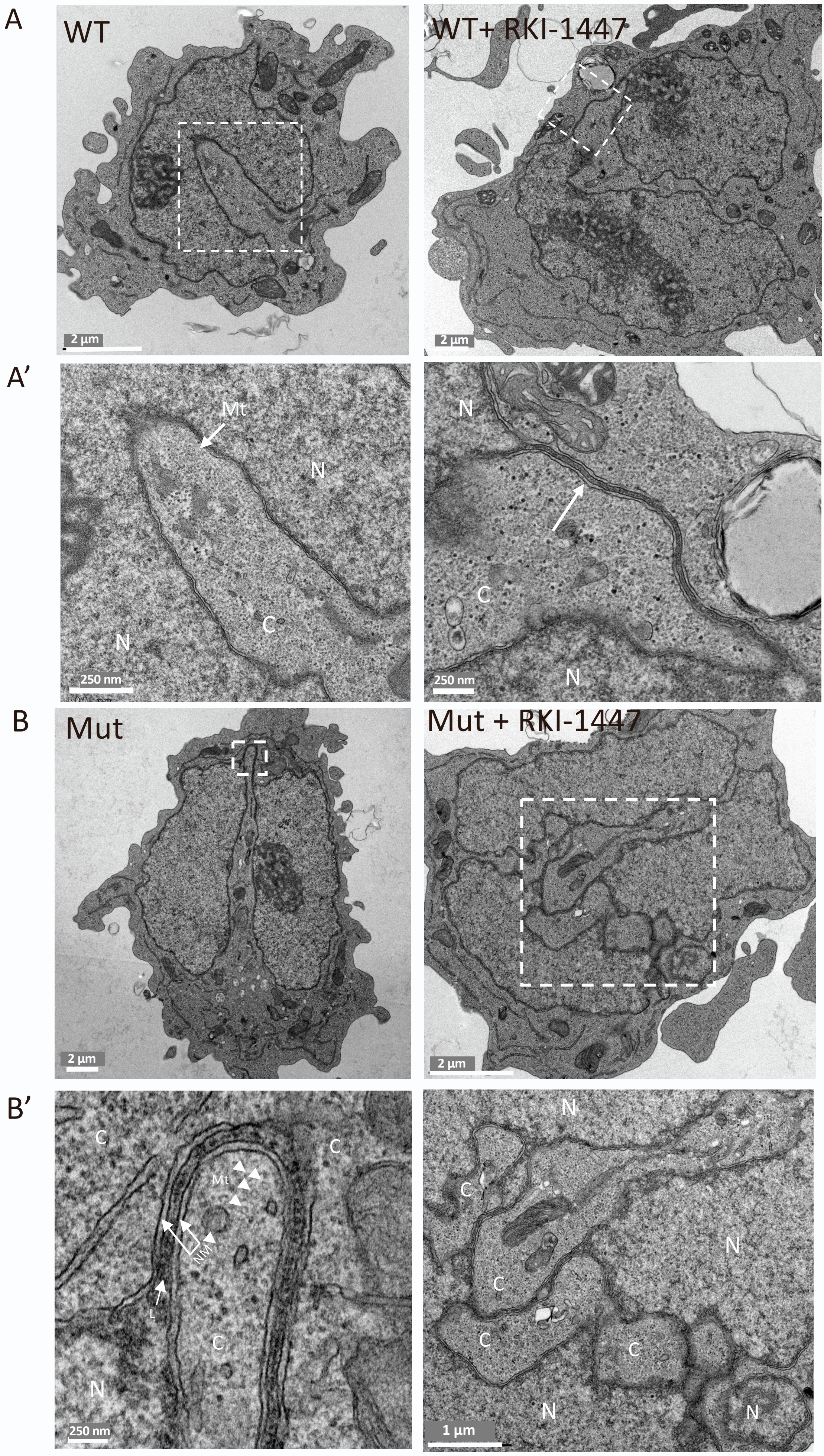
A
WT
WT+ RKI-1447
2 µm
2 µm
A'
Mt
N
N
C
250 nm
N
C
N
250 nm
B
Mut
Mut + RKI-1447
2 µm
2 µm
B'
C
C
Mt
NM
L
C
N
250 nm
N
C
N
C
C
C
N
N
1 µm

Fig. S12: TEM of *SRSF2* WT/Mut MOLM14 cells before and after exposure to RKI-1447. **(A-B)** Showing low magnification images of TEM corresponding to the images shown in **Fig. 4C**. Higher magnification images with rectangles marking the enlarged areas, shown in **A'** and **B'**, respectively. N and C mark nuclear and cytoplasmic areas, respectively. Arrows or arrowheads marked "Mt" point to microtubules, shown in longitudinal or cross sections, respectively. The arrow in **A'**-right points to the inter-lobular sheet, connecting the two nuclear segments. The twin-arrow in **B'**-left (NM) point to the attached nuclear membranes, and the arrow marked "L" point to the nuclear laminae.

Fig. S13

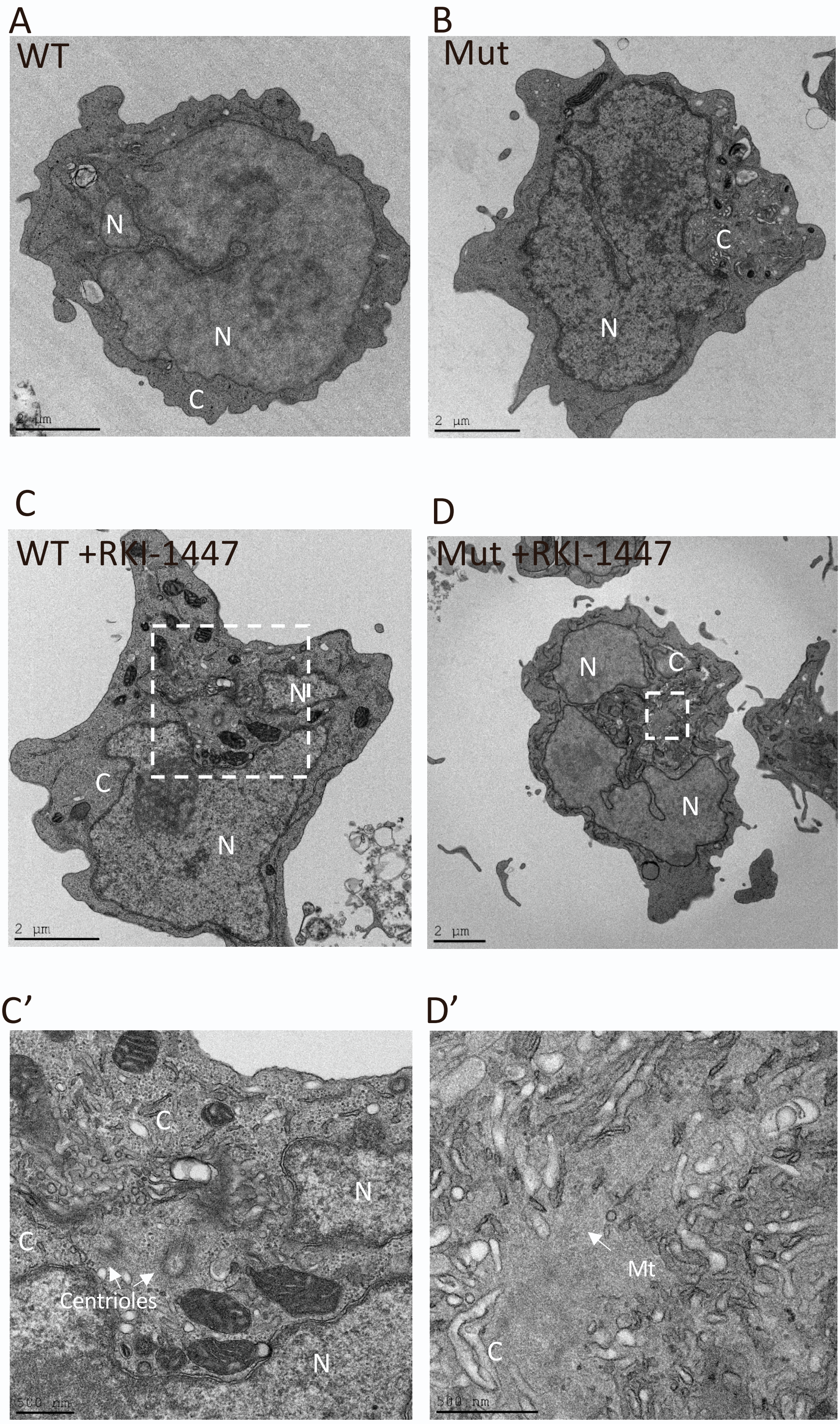

Fig. S13: TEM of *SRSF2* WT /Mut OCI-AML2 cells before and after exposure to RKI-1447. (A-D) Showing lower magnification images of TEM in *SRSF2* WT/Mut OCI-AML2 cells, either untreated or treated with RKI-1447 (0.5µM) for 24hours as indicated. **(C'-D')** Higher magnification images with rectangles marking the enlarged areas, shown in (**C**) and **(D).** N and C mark nuclear and cytoplasmic areas, respectively. Arrows marked "Mt" point to microtubules, shown in longitudinal. The arrow in **C'** and **D'** point to the centrioles or microtubules in the cytoplasm near the nucleus indentation. Note, that this phenomenon are often presented in OCI-AML2 cells after RKI-1447 treatment.

Fig. S14

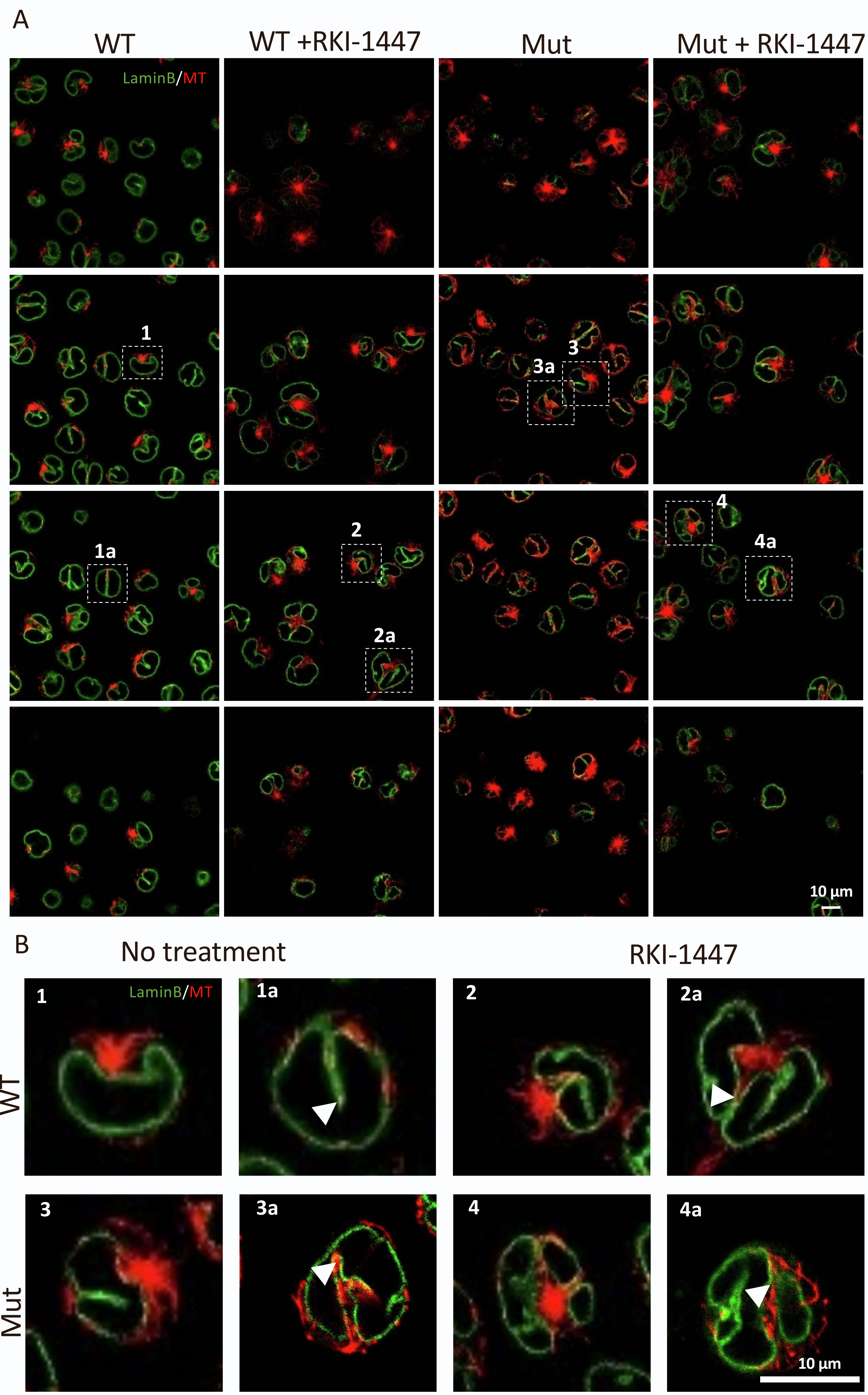
A
WT
WT +RKI-1447
Mut
Mut + RKI-1447
LaminB/MT
1
3
3a
1a
2
4
4a
2a
10 µm
B
No treatment
RKI-1447
WT
Mut
1
1a
2
2a
3
3a
4
4a
LaminB/MT
10 µm

Fig. S14: Confocal images of LaminB1 and microtubules staining of *SRSF2* WT and Mut MOLM14 cells before and after exposure to RKI-1447. *SRSF2* WT/Mut MOLM14 cells were either untreated or 24-hour treated with RKI-1447 (0.5 µM), as indicated. LaminB1 labeling shown in green and microtubules in red. (**A**) Top to bottom rows: single confocal slices bottom to top direction extracted from 3D volume. The depth of the WT volume ranges from 0 to 11.4 µm, with confocal slices shown at intervals: 3.8 µm and 7.6 µm, the Mut volume ranges from 0 to 12.6 µm with confocal image intervals at 4.2 µm and 8.4 µm, WT + RKI-1447 is 12 µm deep with image intervals 4 µm and 8 µm, and Mut + RKI-1447 has a z-depth of 11.1 µm with image intervals at 3.7 µm and 7.4 µm. These are presented in order to give the view of depth of nuclear indentations and degree of lobulation. See also **Movie S1**. (**B**) Selected enlarged insets from images presented in panel (**A**)**.** Cells in (**A**) are outlined with dotted white line, and presented in panel (**B**) according to numbers and treatment conditions. Note the presence of the microtubule organizing center at the base of nuclear deformation – indicated by bright microtubule labeling in first and third columns. Different focal planes reveal deep narrow indentations positive for LaminB1 and containing microtubules as indicated by red labeling (arrowheads in second and fourth columns).

Fig. S15

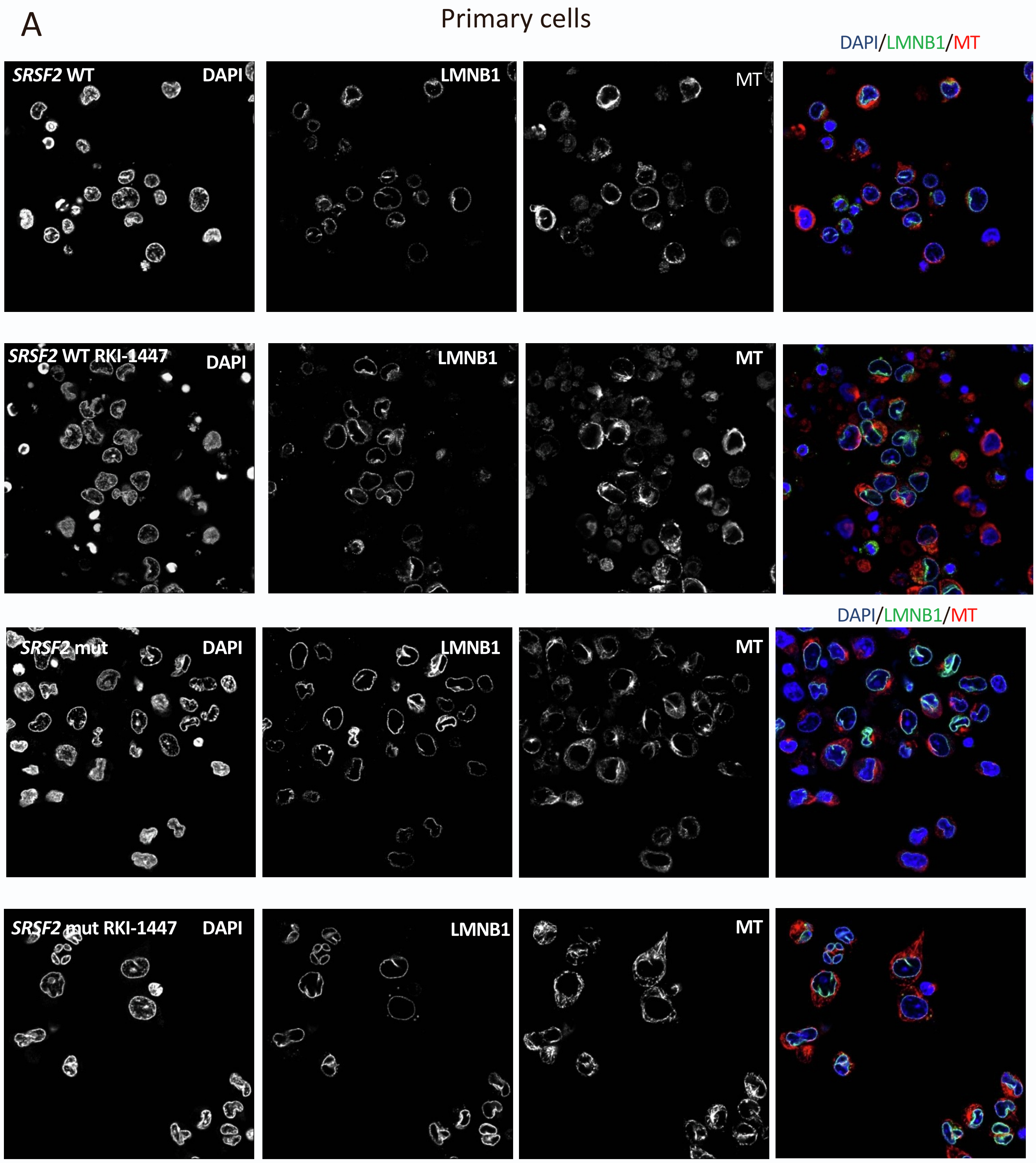

Fig. S15: Confocal images of *SRSF2* Mut/WT primary AML cells before and after exposure to RKI-1447. (A) *SRSF2* WT/Mut primary AML cells, untreated or treated with RKI-1447 (1µM) for 72 hours before fixation, were acquired using the Leica SP8 scanning confocal microscope. Cells were labeled with anti-LaminB1 antibodies to outline nuclear membrane and anti-a-tubulin antibodies to label microtubules, nucleus were labeled with DAPI. Representative confocal slices corresponding to the middle plane of the cells are shown. Sphericity of the nucleus were measured and presented in **Fig. 4B.**

Fig. S16

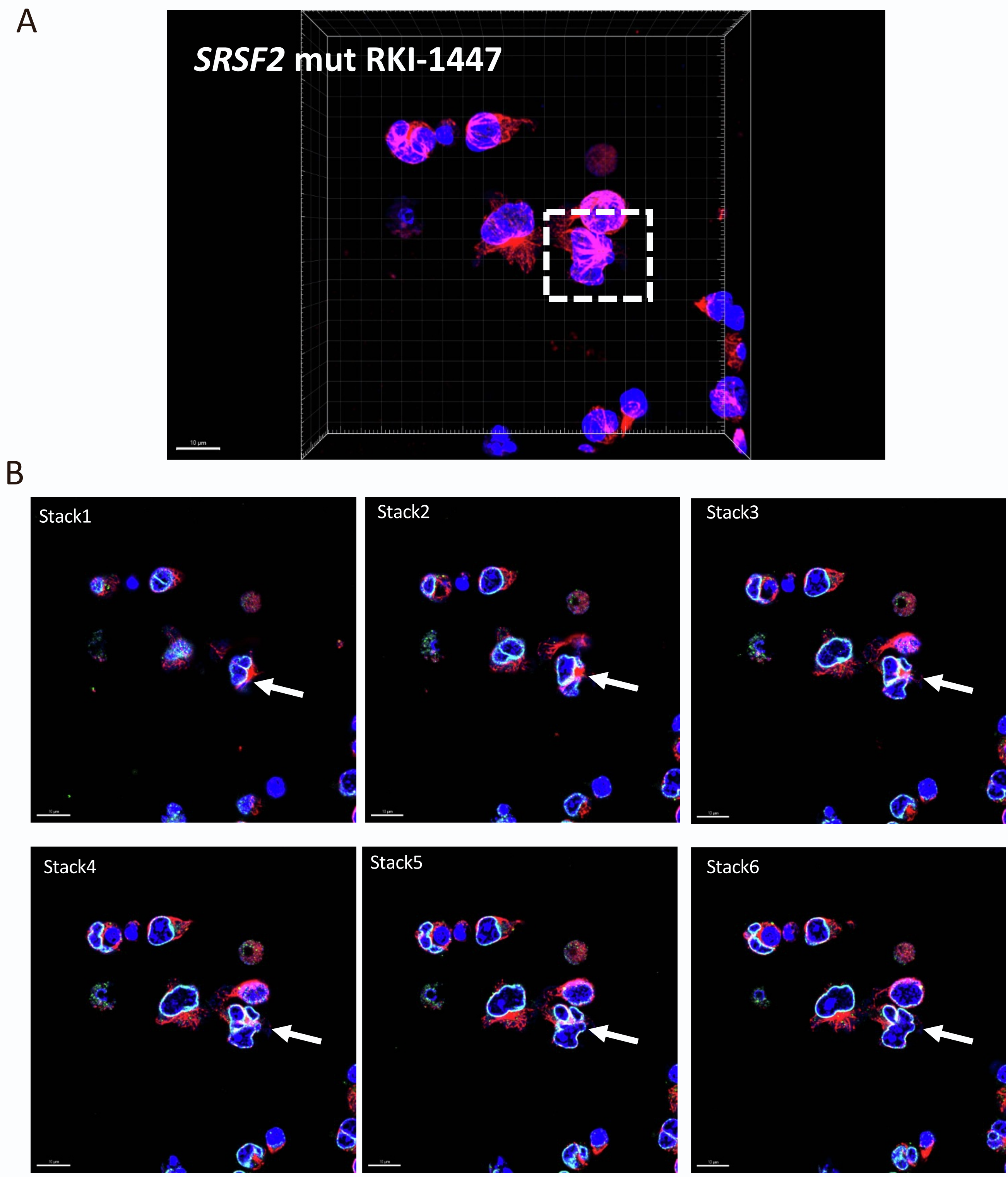

Fig. S16: Representative confocal images of *SRSF2* Mut primary AML cells after exposure to RKI-1447. *SRSF2* Mut primary AML cells were treated with RKI-1447 (1μM) for 72 hours. Cells were labeled with anti-LaminB1 antibodies to outline nuclear membrane (shown in green) and anti-α-tubulin antibodies to label microtubules (shown in red), and nucleus were labeled with DAPI. (**A**) Representative confocal slice corresponding to the middle plane of the cells is shown. (**B**) Different Z-stacks of the highlighted cells in (**A**) were shown. Note, deep narrow folds or invagination of nuclear membrane that are presented upon RKI-1447 treatment. Red signal detected inside such invaginations points to the presence of microtubules (arrows).

Fig. S17

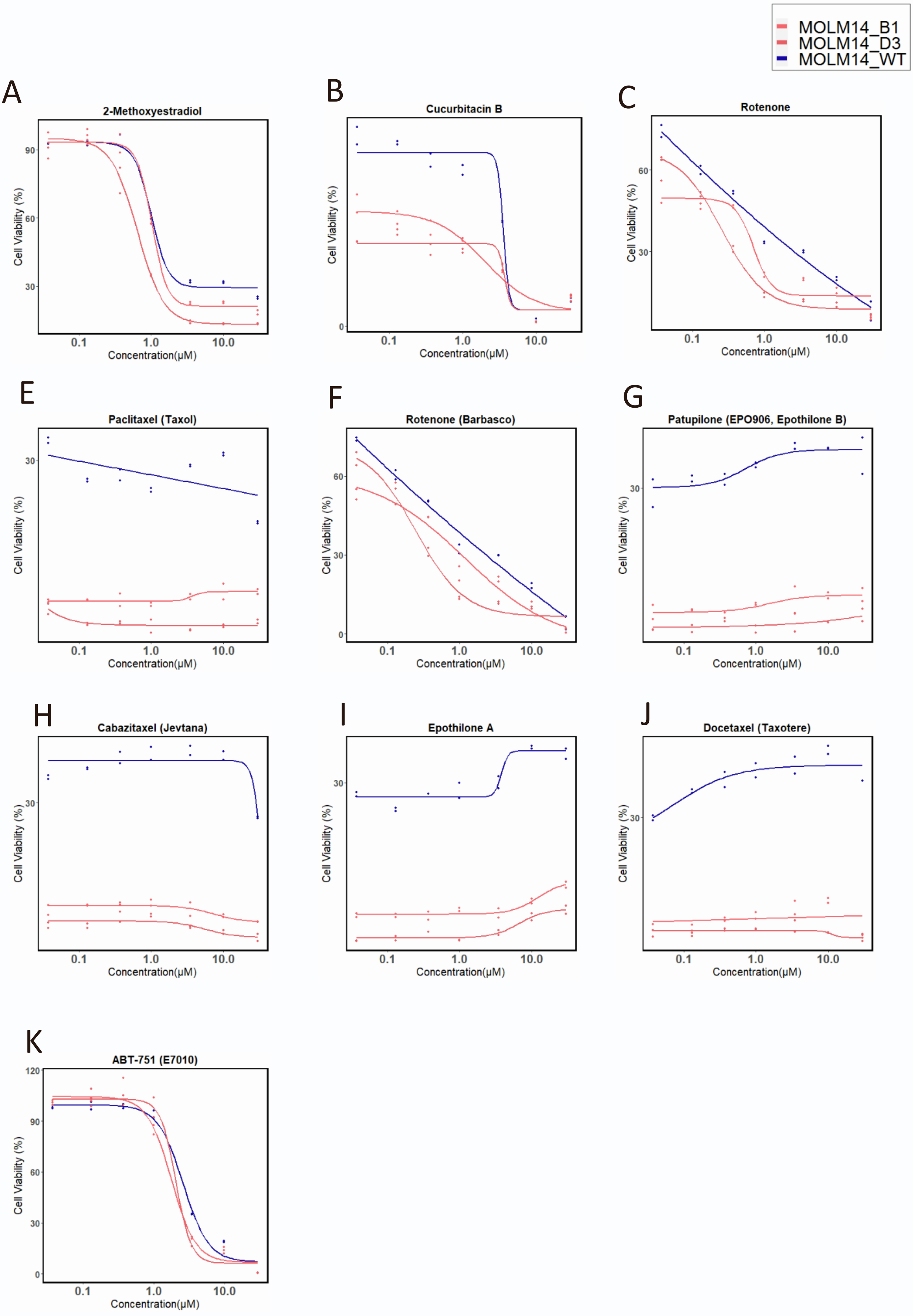

Fig. S17: Dose response of some cytoskeleton compounds in *SRSF2* WT and Mut MOLM14 cells. Cell viability of *SRSF2* WT and two *SRSF2* Mut MOLM14 cell lines (B1 and D3) were measured after 48 hours exposure to cytoskeletal compounds at concentration of 0.037μM, 0.129μM, 0.369μM, 0.99 μM, 3.49μM, 9.97μM and 29.90μM. Names of compounds are indicated in the title of the plot. The highest three concentrations were compared between WT and Mutated clones by two-way ANOVA, followed by Dunnett’s post-hoc test, see also **Data files S10** .
